# Context-dependent effects of *CDKN2A* and other 9p21 gene losses during the evolution of esophageal cancer

**DOI:** 10.1038/s43018-024-00876-0

**Published:** 2025-01-03

**Authors:** Piyali Ganguli, Celia C. Basanta, Amelia Acha-Sagredo, Hrvoje Misetic, Maria Armero, Akram Mendez, Aeman Zahra, Ginny Devonshire, Gavin Kelly, Adam Freeman, Mary Green, Emma Nye, Anita Bichisecchi, Paola Bonfanti, Oesophageal Cancer Clinical and Molecular Stratification (OCCAMS) Consortium, Rebecca C. Fitzgerald, Paul A. W. Edwards, Nicola Grehan, Barbara Nutzinger, Aisling M. Redmond, Christine Loreno, Sujath Abbas, Adam Freeman, Elizabeth C. Smyth, Maria O’Donovan, Ahmad Miremadi, Shalini Malhotra, Monika Tripathi, Hannah Coles, Curtis Millington, Matthew Eldridge, Maria Secrier, Ginny Devonshire, Jim Davies, Charles Crichton, Nick Carroll, Richard H. Hardwick, Peter Safranek, Andrew Hindmarsh, Vijayendran Sujendran, Stephen J. Hayes, Yeng Ang, Andrew Sharrocks, Shaun R. Preston, Izhar Bagwan, Vicki Save, Richard J. E. Skipworth, Ted R. Hupp, J. Robert O’Neill, Olga Tucker, Andrew Beggs, Philippe Taniere, Sonia Puig, Gianmarco Contino, Timothy J. Underwood, Robert C. Walker, Ben L. Grace, Jesper Lagergren, James Gossage, Andrew Davies, Fuju Chang, Ula Mahadeva, Vicky Goh, Francesca D. Ciccarelli, Grant Sanders, Richard Berrisford, David Chan, Ed Cheong, Bhaskar Kumar, L. Sreedharan, Simon L. Parsons, Irshad Soomro, Philip Kaye, John Saunders, Laurence Lovat, Rehan Haidry, Michael Scott, Sharmila Sothi, Suzy Lishman, George B. Hanna, Christopher J. Peters, Krishna Moorthy, Anna Grabowska, Richard Turkington, Damian McManus, Helen Coleman, Russell D. Petty, Freddie Bartlett, Manuel Rodriguez-Justo, Jo Spencer, Rebecca C. Fitzgerald, Francesca D. Ciccarelli

**Affiliations:** 1 Cancer Systems Biology Laboratory, The Francis Crick Institute, London, UK; 2 Barts Cancer Institute - Centre for Cancer Evolution, Queen Mary University of London, London, UK; 3 Early Cancer Institute, Hutchison Research Centre, University of Cambridge, Cambridge, UK; 4 Bioinformatics & Biostatistics STP, The Francis Crick Institute, London, UK; 5 Experimental Histopathology STP, The Francis Crick Institute, London, UK; 6 Epithelial Stem Cell Biology & Regenerative Medicine Laboratory, The Francis Crick Institute, London, UK; 7 Institute of Immunity & Transplantation, Division of Infection & Immunity, UCL, London, UK; 8 Department of Pathology, UCL Cancer Institute, London, UK; 9 School of Immunology and Microbial Sciences, King’s College London, London, UK; 10 Early Cancer Institute, University of Cambridge, Cambridge, UK; 11 Cancer Research UK Cambridge Institute, University of Cambridge, Cambridge, UK; 12 Cambridge University Hospitals NHS Foundation Trust, Cambridge, UK; 13 Department of Histopathology, Addenbrooke’s Hospital, Cambridge, UK; 14 Department of Computer Science, University of Oxford, Oxford, UK; 15 Salford Royal NHS Foundation Trust, Salford, UK; 16 Faculty of Medical and Human Sciences, University of Manchester, Manchester, UK; 17 Wigan and Leigh NHS Foundation Trust, Wigan, UK; 18 GI Science Centre, University of Manchester, Manchester, UK; 19 Royal Surrey County Hospital NHS Foundation Trust, Guildford, UK; 20 Edinburgh Royal Infirmary, Edinburgh, UK; 21 Edinburgh University, Edinburgh, UK; 22 University Hospitals Birmingham NHS Foundation Trust, Birmingham, UK; 23 Heart of England NHS Foundation Trust, Birmingham, UK; 24 Institute of Cancer and Genomic Sciences, University of Birmingham, Birmingham, UK; 25 University Hospital Southampton NHS Foundation Trust, Southampton, UK; 26 Cancer Sciences Division, University of Southampton, Southampton, UK; 27 Guy’s and St Thomas’s NHS Foundation Trust, London, UK; 28 Karolinska Institute, Stockholm, Sweden; 29 Barts Cancer Institute, Queen Mary University of London, London, UK; 30 Plymouth Hospitals NHS Trust, Plymouth, UK; 31 Norfolk and Norwich University Hospitals NHS Foundation Trust, Norwich, UK; 32 Nottingham University Hospitals NHS Trust, Nottingham, UK; 33 University College London, London, UK; 34 Wythenshawe Hospital, Manchester, UK; 35 University Hospitals Coventry and Warwickshire NHS Trust, Coventry, UK; 36 Peterborough Hospitals NHS Trust, Peterborough City Hospital, Peterborough, UK; 37 Department of Surgery and Cancer, Imperial College, London, UK; 38 Queen’s Medical Centre, University of Nottingham, Nottingham, UK; 39 Centre for Cancer Research and Cell Biology, Queen’s University Belfast, Belfast, Northern Ireland; 40 Tayside Cancer Centre, Ninewells Hospital and Medical School, Dundee, Scotland; 41 Portsmouth Hospitals NHS Trust, Portsmouth, UK

**Keywords:** Cancer genomics, Oesophageal cancer, Tumour-suppressor proteins, Cancer

## Abstract

*CDKN2A* is a tumor suppressor located in chromosome 9p21 and frequently lost in Barrett’s esophagus (BE) and esophageal adenocarcinoma (EAC). How *CDKN2A* and other 9p21 gene co-deletions affect EAC evolution remains understudied. We explored the effects of 9p21 loss in EACs and cancer progressor and non-progressor BEs with matched genomic, transcriptomic and clinical data. Despite its cancer driver role, *CDKN2A* loss in BE prevents EAC initiation by counterselecting subsequent *TP53* alterations. 9p21 gene co-deletions predict poor patient survival in EAC but not BE through context-dependent effects on cell cycle, oxidative phosphorylation and interferon response. Immune quantifications using bulk transcriptome, RNAscope and high-dimensional tissue imaging showed that *IFNE* loss reduces immune infiltration in BE, but not EAC. Mechanistically, *CDKN2A* loss suppresses the maintenance of squamous epithelium, contributing to a more aggressive phenotype. Our study demonstrates context-dependent roles of cancer genes during disease evolution, with consequences for cancer detection and patient management.

## Abstract (web summary)

Ganguli et al. show that *CDKN2A* loss in Barrett’s esophagus prevents esophageal adenocarcinoma initiation by counterselecting subsequent TP53 loss and report context-dependent effects of 9p21 gene co-deletions on disease progression.

## Main

*CDKN2A* is among the most frequently damaged cancer genes, with loss of function (LoF) reported in at least 35 different tumor types across 12 organ systems^1^. *CDKN2A* acts as a tumor suppressor by inducing cell cycle arrest and cellular senescence^2^ as well as preventing angiogenesis^3^, oxidative stress^4^, and metastasis^2^. Additionally, *CDKN2A* LoF predicts poor patient survival^5–7^.

**Fig. 1:**
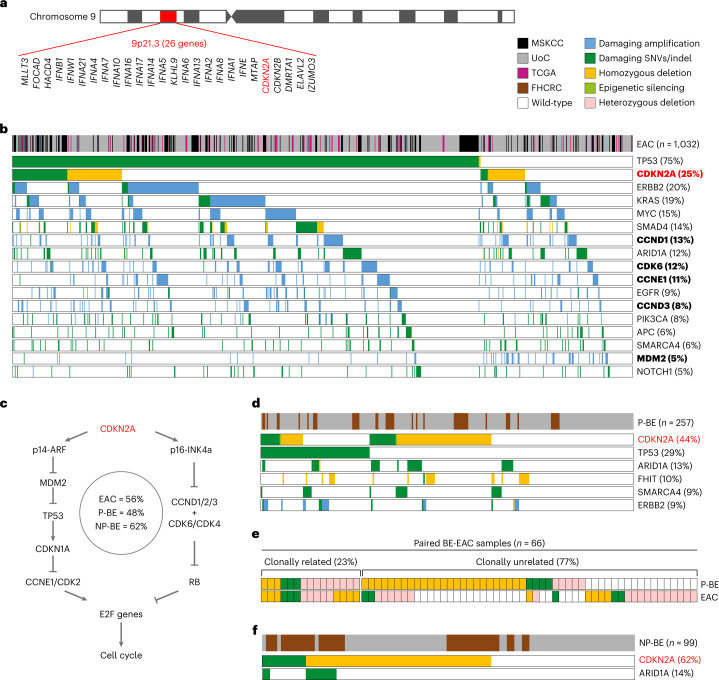
*CDKN2A* LoF occurrence in BE and EAC. **a**, Gene composition of chromosome 9p21 locus. **b**, Canonical EAC drivers damaged in at least 5% of EACs (*n* = 1,032 patients). All cell cycle regulators are reported in bold. **c**, Alterations in cell cycle regulators in BE and EAC. *CDKN2A* gene products (p14-ARF and p16-INK4a) regulate the cell cycle through the E2F genes^93^. p14-ARF blocks MDM2 and TP53 degradation, which induces *CDKN1A* transcription. CDKN1A in turn inhibits the CCNE1/CDK2 complex ultimately blocking cell cycle through E2F1 inhibition. p16-INK4a directly inhibits the CCND/CDK6/CDK4 complex preventing RB1 phosphorylation. Unphosphorylated RB1 can bind E2F1, leading to cell cycle arrest. *CDKN2A* LoF favors cell cycle progression resulting in uncontrolled cell proliferation. Values within the circle represent the proportion of EACs, P-BEs and NP-BEs with at least one damaged cell cycle regulator (except TP53). **d**, Canonical EAC drivers damaged in at least 5% of P-BEs (*n* = 257 patients). **e**, Paired BE-EACs (*n* = 66 patients) with *CDKN2A* LoF. Clonally related alterations refer to either identical *CDKN2A* alterations in both lesions or *CDKN2A* alterations in BE that could further evolve in EAC. **f**, Canonical drivers damaged in at least 5% of NP-BEs (*n* = 99 patients). Alteration frequency of EAC canonical drivers in **b**, **d** and **f** is indicated in brackets. The alteration frequency of all EAC drivers in the three cohorts is available in Supplementary Table 2. FHCRC, Fred Hutchinson Cancer Research Center; LoF, loss of function; MSKCC, Memorial Sloan Kettering Cancer Center; NP-BE, non-progressor Barrett’s esophagus; EAC, esophageal adenocarcinoma; P-BE, progressor Barrett’s esophagus; SNV, single-nucleotide variant; TCGA, The Cancer Genome Atlas; UoC, University of Cambridge. Source data

*CDKN2A* LoF may occur through damaging point mutations, small indels or large deletions of chromosome 9p21.3 locus (hereon 9p21), an event observed in around 15% of cancers^8^. Depending on their length, 9p21 deletions may involve up to 26 genes, including other cell cycle regulators (*CDKN2B* and *KLHL9*), a metabolic enzyme (*MTAP*) and a cluster of 16 type I interferons (Fig. 1a). Recently, the loss of the whole locus, rather than *CDKN2A* alone, has been associated with poor survival and resistance to immunotherapy, possibly through the onset of an immune-cold tumor microenvironment (TME)^8^.

Dissecting the consequences of individual 9p21 gene losses is not straightforward because of their co-occurrence. Recently, the induction of different 9p21 deletions in pancreatic cancer mouse models enabled observation of reduced CD8^+^ T cell infiltration only when the IFN cluster was co-deleted with *CDKN2A*, *CDKN2B* and *MTAP*^9^. *IFNE*, one of the 9p21 type-I interferons (Fig. 1a), is a tumor suppressor in ovarian cancer^10^, and IFNE treatment promotes CD8^+^ T cell activation while reducing T regulatory cells (T_reg_ cells) and myeloid-derived suppressor cells (MDSCs)^10^. Also, *MTAP* can regulate CD8^+^ and CD4^+^ T cell infiltration in melanoma mouse models by controlling methylthioadenosine accumulation in their TME^11^. These studies started to unveil that at least some of the effects previously ascribed to *CDKN2A* LoF are in fact due to the loss of other 9p21 genes.

*CDKN2A* LoF has long been known as an early event in the evolution of esophageal adenocarcinoma (EAC), occurring already in its precursor, Barrett’s esophagus (BE)^12–16^. Consequently, *CDKN2A* LoF has been proposed to drive EAC initiation by favoring BE clonal selective sweeps and subsequent alterations of additional drivers, most frequently *TP53* (refs. ^17–20^). Recently, this model has been replaced by an alternative one where early *TP53* LoF would enable whole-genome doubling with consequent acquisition of additional drivers^21,22^. The role of *CDKN2A* LoF in EAC initiation remains controversial. Some studies reported higher frequency of *CDKN2A* LoF in BE cases progressing to EAC compared to BEs that did not progress^23–27^, implying that *CDKN2A* inactivation favors cancer initiation. Other studies found either no difference between progressor and non-progressor BEs^22,28–31^ or a higher frequency of *CDKN2A* LoF in non-progressor BEs^15^. This uncertainty raises questions on the role of *CDKN2A* in BE and EAC evolution. Moreover, very little is known about the function of the remaining 9p21 genes.

Here, we investigated how the loss of *CDKN2A* and other 9p21 genes affects EAC initiation and progression. We compared genomic, transcriptomic and survival data from large and clinically annotated cohorts of EAC and patients with BE who progressed or did not progress to cancer. We validated the results in vitro and studied the effect of 9p21 loss on BE and EAC TME by high-dimensional tissue profiling coupled with RNAscope. Finally, we rebuilt the causal gene regulatory networks linking *CDKN2A* gene loss to specific downstream functional effects. Our results suggested that the same genetic alterations of *CDKN2A* and other 9p21 genes have different effects in different contexts and stages of EAC evolution, with possible implications in patient management.

## Results

### *CDKN2A* LoF drives BE and EAC evolution, but not EAC initiation

**Extended Data Fig. 1:**
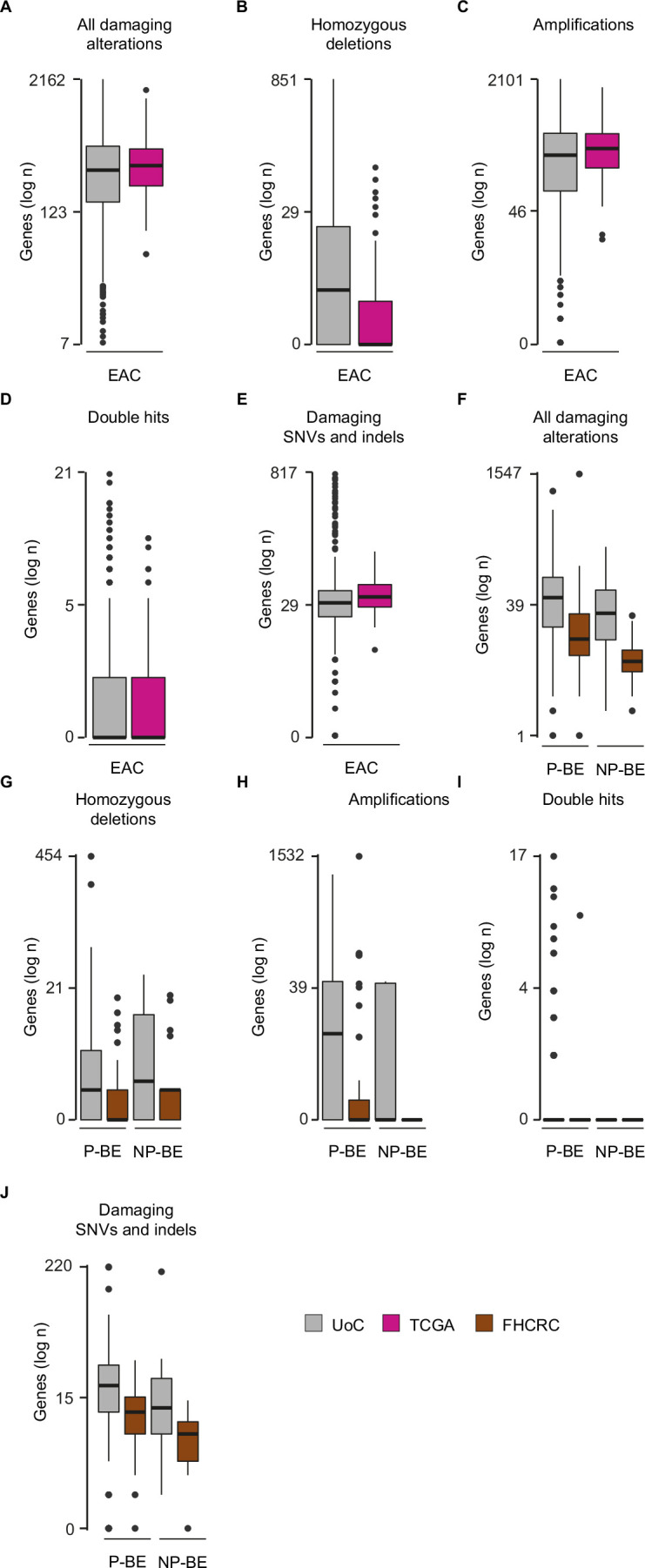
Distribution of damaged genes in BE and EAC. Damaged genes per sample in EAC WGS/WES cohorts (n = 779 patients) with any type of damaging alterations (**A**), homozygous deletions (**B**), gene amplifications (**C**), double hits (**D**) and damaging SNVs and indels (**E**). Number of damaged genes per sample in P-BE (n = 218 patients) and NP-BE (n = 63 patients) with any type of damaging alterations (**F**), homozygous deletions (**G**), gene amplifications (**H**), double hits (**I**) and damaging SNVs and indels (**J**). FHCRC, Fred Hutchinson Cancer Research Center; NP-BE, non-progressor Barrett’s esophagus; EAC, esophageal adenocarcinoma; P-BE, progressor Barrett’s esophagus; SNVs, single nucleotide variants; TCGA, The Cancer Genome Atlas; UoC, University of Cambridge. All boxplots show first and third quartiles, whiskers extend to 1.5X the interquartile lower and upper range and the line indicates the median. Source data

We collected whole-genome sequencing (WGS), whole-exome sequencing (WES) and gene panel sequencing data for 1,032 EACs from the literature^6,32–38^ or sequenced *de novo* by the Esophageal Cancer Clinical and Molecular Stratification (OCCAMS) Consortium (Supplementary Table 1). Our cohort reflected EAC high male prevalence, with almost 9:1 male-to-female incidence ratio^39^ (Supplementary Table 1). To ensure consistency, we annotated damaging mutations and copy-number alterations in all datasets using the same approach (Methods and Extended Data Fig. 1a–e). Because *CDKN2A* can be silenced also via epigenetic modifications, we analyzed methylation data for a subset of EACs^32,40^ (Supplementary Table 1). We then identified the damaged drivers in each sample using a curated list of 54 known (canonical) EAC drivers (Supplementary Table 2). In agreement with previous studies^29,32,41^, *CDKN2A* was the second most frequently damaged EAC driver, with LoF in 25% of samples (Fig. 1b). More than 56% of EACs (90% considering also *TP53*) had damaging alterations in other cell cycle regulators (Fig. 1c and Supplementary Table 2), suggesting that cell cycle disruption is key in EAC evolution but does not always involve *CDKN2A*.

Next, we measured the frequency of *CDKN2A* LoF in 257 BEs that progressed to high-grade dysplasia or EAC (P-BEs), again sequenced for this study or gathered from published datasets^15,40,42–44^ (Supplementary Table 1 and Extended Data Fig. 1f–j). *CDKN2A* LoF occurred significantly more frequently in P-BE than EAC (*P* = 4 × 10^−9^, two-sided Fisher’s exact test; Fig. 1d), suggesting that EAC does not always originate from a *CDKN2A*-damaged BE. To further investigate this, we analyzed 66 matched EAC-BE pairs with *CDKN2A* LoF in BE or EAC (Supplementary Table 1). Only 15 matched lesions had either identical or clonally related *CDKN2A* alterations (Fig. 1e), confirming that *CDKN2A* LoF is not required for precancer to cancer transition. Interestingly, 28 EACs lost *CDKN2A* independently of the paired BEs (Fig. 1e), suggesting that either EAC developed from a different *CDKN2A*-damaged BE clone or *CDKN2A* LoF was acquired after transformation.

Finally, we analyzed 99 BEs that did not progress to high-grade dysplasia or EAC (NP-BEs)^15,40,43,44^ (Supplementary Table 1 and Extended Data Fig. 1f–j). The frequency of *CDKN2A* LoF in NP-BE was even higher than P-BE and EAC (*P* = 3 × 10^−3^ and *P* = 3 × 10^−13^, respectively, two-sided Fisher’s exact test, Fig. 1f). Moreover, although in EAC, the dysregulation of cell cycle could occur through alterations of other genes, *CDKN2A* was the only gene encoding a cell cycle regulator damaged in BE (Fig. 1d). Therefore, unlike EAC, only *CDKN2A* LoF is relevant for BE evolution.

As observed previously^22,45^, P-BEs had significantly more damaged drivers than NP-BEs (*P* = 7 × 10^−6^, two-sided Fisher’s exact test; Supplementary Table 2), indicating that EAC initiation requires several driver events, most frequently *TP53* complete loss. Given its high recurrence, we used *TP53* LoF to assess the role of *CDKN2A* LoF in EAC initiation calculating the odds of cancer progression based on the mutational status of *CDKN2A* and *TP53* in BE. As expected, the odds of cancer progression in BE cases with *TP53* LoF was 1 irrespective of *CDKN2A* status (Supplementary Table 3), confirming that *TP53* is a strong driver of EAC initiation. However, the odds of cancer progression in BEs with *CDKN2A* LoF and wild-type *TP53* was lower than those of BEs with both wild-type genes (0.58 and 0.72, respectively; Supplementary Table 3). This suggested that an early occurrence of *CDKN2A* LoF in BE may reduce the likelihood of EAC initiation. To test this further, we compared two logistic regression models, one assuming a role in EAC initiation only for *TP53* LoF (model 1) and the other for both *TP53* and *CDKN2A* LoFs (model 2; Methods). Model 2 was a significantly better predictor of EAC initiation than model 1 (*P* = 0.01, ANOVA test), with expected occurrences of P-BEs with any status of *TP53* and *CDKN2A* perfectly matching the observed occurrences (Supplementary Table 3). The negative β coefficient of *CDKN2A* in model 2 further confirmed that *CDKN2A* LoF may reduce risk of cancer progression (Methods and Supplementary Table 3).

### *TP53* loss reduces proliferation of *CDKN2A* LoF BE cells

**Fig. 2:**
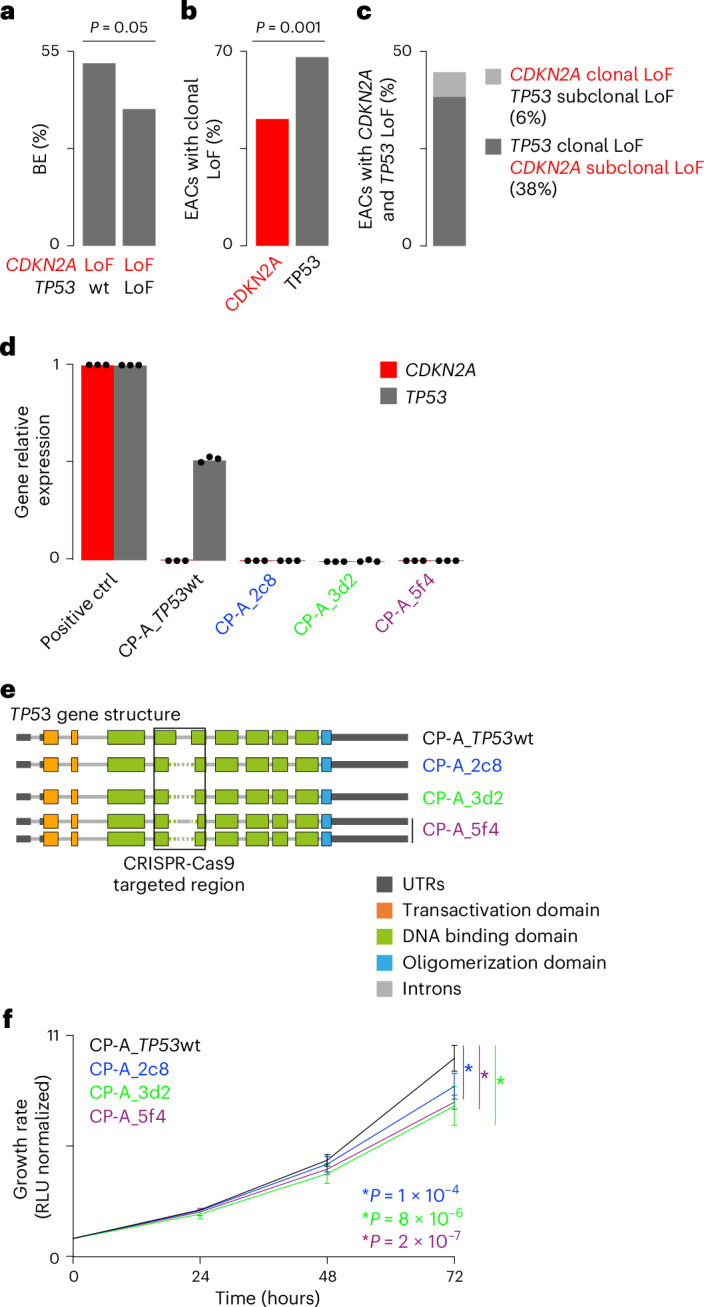
Effect of *TP53* loss in BE with *CDKN2A* LoF. **a**, Frequency of *CDKN2A* LoF in 356 BEs (*n* = 257 P-BE and *n* = 99 NP-BE individuals, respectively) with or without *TP53* LoF. Statistical significance was assessed using a two-sided Fisher’s exact test (*P* = 0.05). **b**, Frequency of EACs with clonal LoF alterations in *CDKN2A* and *TP53* genes. For this analysis, *n* = 580/779 patients with EAC with WGS or WES data and LoF in these genes were considered. Statistical significance was assessed using a two-sided Fisher’s exact test (*P* = 0.001). **c**, Frequency of EACs with clonal and subclonal LoF alterations in *CDKN2A* and *TP53* genes in *n* = 47 patients with WGS or WES data and damaging alterations in both genes. **d**, *CDKN2A* and *TP53* gene expression levels quantified by RT–qPCR of RNA from *TP53* wild-type CP-A cells (CP-A_*TP53*wt), three *TP53* KO clones (CP-A_2c8, CP-A_3d2, CP-A_5f4) and control RNA relativized to *ACTB* expression. One biological replicate was performed with three technical replicates. **e**, *TP53* gene structure in CP-A_*TP53*wt, CP-A_2c8, CP-A_3d2 and CP-A_5f4 cells. Exon-intron arrangement was derived from the UCSC genome browser (https://genome.ucsc.edu/) based on NM_000546 mRNA sequence (chr17:7,668,421-7,687,490, hg38 assembly). Dotted lines represent edited regions. **f**, Growth curves of CP-A_*TP53*wt, CP-A_2c8, CP-A_3d2 and CP-A_5f4 cells. Proliferation was assessed every 24 h and normalized to time zero. Mean values at 72 h were compared by two-tailed Student’s *t*-test (*P* = 1 × 10^−4^, 8 × 10^−6^ and 2 × 10^−7^, respectively). Error bars show standard deviation. Three biological replicates were performed, each in two to four technical replicates. ctrl, control; KO, knockout; P-BE, progressor Barrett’s esophagus; RLU, relative light unit; RT–qPCR, real-time quantitative PCR; RQ, relative quantification; UTR, untranslated region; wt, wild type. Source data

Next, we set out to investigate how *CDKN2A* LoF in BE could prevent EAC initiation. As the proportion of BEs with both *CDKN2A* and *TP53* LoF was significantly lower than that of BEs with *CDKN2A* LoF only (*P* = 0.05, two-sided Fisher’s exact test; Fig. 2a), we hypothesized that negative selection might act on BE cells losing both genes. To test this hypothesis, we compared *CDKN2A* and *TP53* LoF clonality in 580 EACs with WGS or WES data, as clonality informs on when alterations are acquired during cancer evolution. Despite the well-known EAC intratumor heterogeneity^14^, *CDKN2A* or *TP53* LoFs were clonal in almost 70% of EACs (397/580), confirming that both alterations are early events. However, EACs with fully clonal *CDKN2A* LoF were significantly fewer than those with fully clonal *TP53* LoF (*P* = 0.001, two-sided Fisher’s exact test; Fig. 2b), suggesting that overall*TP53* LoF tends to predate *CDKN2A* LoF. In support of this, *CDKN2A* LoF occurred before *TP53* LoF in only 6% of the 47 EACs with LoF alterations in both genes as compared to 38% where *TP53* LoF occurred before that of *CDKN2A* (Fig. 2c). This finding confirmed that the subsequent loss of *TP53* in the presence of *CDKN2A* LoF is a rare event, suggesting that it might be selected against.

Interestingly, BAR-T cells, derived from BE with constitutive loss of *CDKN2A*, increase cell doubling times upon *TP53* knockdown^46^, supporting the hypothesis that the additional loss of *TP53* reduces cell growth rate. To test this experimentally, we induced *TP53* knockout (KO) in metaplastic BE CP-A cells derived from a male individual with *CDKN2A* LoF and wild-type *TP53* (ref. ^47^). First, we confirmed that CP-A cells expressed *TP53* but did not express *CDKN2A* (Fig. 2d). We then used CRISPR-Cas9 to edit *TP53* (Supplementary Table 4) and performed single cell cloning to expand cell colonies. To control for off target effects and clonal differences, we selected three clones with a partial deletion of *TP53* exons 5 and 6 (Fig. 2e), as assessed via amplicon sequencing (Supplementary Table 4). We confirmed that these clones did not express *CDKN2A* nor *TP53* (Fig. 2d). The fact that we could isolate clones losing both genes implied that BE cells with *CDKN2A* LoF can survive subsequent *TP53* loss. However, compared to *TP53* wild-type CP-A cells, all three *TP53* KO CP-A clones showed significantly slower growth rate that was already visible after 72 h (two-sided *t*-test test, Fig. 2f).

This finding was in line with the reported increase in cell doubling times of *TP53* knockdown BAR-T cells^46^ and supported the tumor-preventive role of early *CDKN2A* inactivation due to the reduced fitness, defined as proliferative capacity, of cells additionally losing *TP53*.

### LoF of 9p21 genes predicts poor survival in EAC, but not in BE

**Fig. 3:**
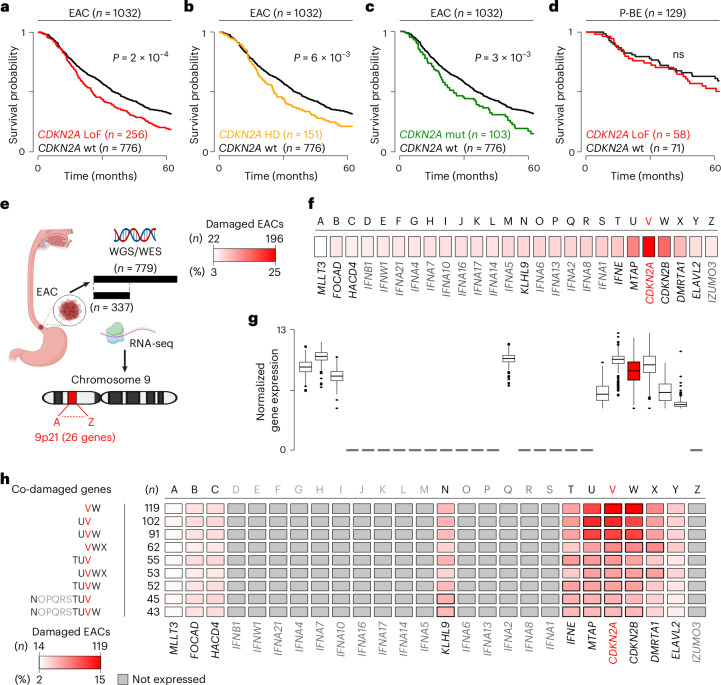
Effect of the LoF of *CDKN2A* and other 9p21 genes on survival. **a**–**c**, Kaplan–Meier survival curves of *n* = 1,032 patients with EAC with wild-type *CDKN2A* compared to those with all types of LoFs (*P* = 2 × 10^−4^) (**a**), only homozygous deletions (*P* = 6 × 10^−3^) (**b**) and only LoF mutations (*P* = 3 × 10^−3^) (**c**). **d**, Kaplan–Meier survival curves of *n* = 129 patients with P-BE with and without *CDKN2A* LoF. Log-rank method was used to estimate the *P* values. ns, not significant. **e**, Approach to test the effect of the co-damage in 9p21 genes on patient survival. Only *n* = 779 patients with EAC with WGS or WES data were used for the survival analysis, whereas *n* = 337 patients with RNA-seq data were used to measure 9p21 gene expression. Letters correspond to the 26 genes according to their order in the chromosomal locus. **f**, LoF frequency of 9p21 genes in *n* = 779 patients with EAC. **g**, Distribution of normalized expression values in the 9p21 genes in *n* = 337 patients with EAC. Boxplot shows first and third quartiles, whiskers extend to the lowest and highest value within the 1.5× interquartile range and the line indicates the median. **h**, Kaplan–Meier survival analysis of patients with EAC with co-alterations in the ten expressed 9p21 genes and *n* = 413 patients with EAC with a wild-type locus. Only groups with significantly poor survival (FDR < 0.1) are shown and genes of interest are outlined in black. All groups used in the analysis are listed in Supplementary Table 5. The minimum and maximum number and percent of damaged EACs in **f** and **h** are reported in the corresponding heatmap. HD, homozygous deletion; WES, whole-exome sequencing; WGS, whole-genome sequencing. Cartoon in (**e**) was created with BioRender.com. Source data

**Extended Data Fig. 2:**
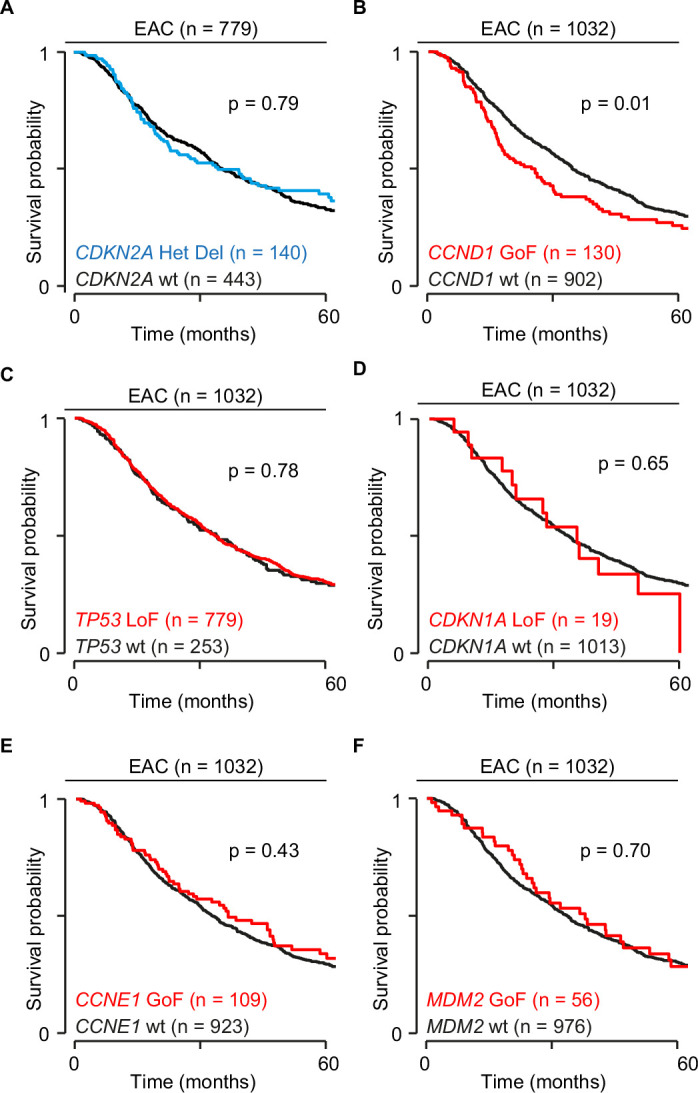
Effect of alterations in cell cycle regulators on EAC survival. **A**. Kaplan-Meier survival curves of patients with EAC with wild type *CDKN2A* compared to those with heterozygous loss of *CDKN2A*. Heterozygous deletions could be inferred only in n = 779 patients with EAC with WGS or WES data. Kaplan-Meier survival curves of patients with EAC with alterations in *CCND1* (p-value = 0.01) **(B)**, *TP53* **(C)**, *CDKN1A* **(D)**, *CCNE1* **(E)** and *MDM2* **(F)** compared to the corresponding wild type samples. Survival curves B-F were done using the whole cohort of 1032 EACs. GoF, gain-of-function; LoF, loss-of-function; EAC, esophageal adenocarcinoma; Het, Heterozygous deletions. Log-rank method was used to estimate the p-values. Source data

Because *CDKN2A* LoF has been associated with poor patient survival^5–7^, we investigated the survival effect of *CDKN2A* and other 9p21 gene LoF in our extended BE and EAC cohorts. Patients with EAC and *CDKN2A* LoF showed significantly worse survival than those with the wild-type gene (Fig. 3a). This difference held true even when patients with *CDKN2A* homozygous deletions (Fig. 3b) or damaging mutations (Fig. 3c) were considered separately. However, we did not observe lower survival in patients with *CDKN2A* heterozygous deletions only (Extended Data Fig. 2a), suggesting that *CDKN2A* complete loss is required to affect prognosis. Damaging alterations in *TP53* or other cell cycle regulators had no effect on survival (Extended Data Fig. 2b–f) despite their frequent EAC alterations (Fig. 1c). Therefore, the survival effect of *CDKN2A* LoF does not depend on its function as cell cycle regulator. Moreover, *CDKN2A* LoF was not a predictor of worse survival in P-BE (Fig. 3d), again suggesting context-dependent consequences of its loss.

**Extended Data Fig. 3:**
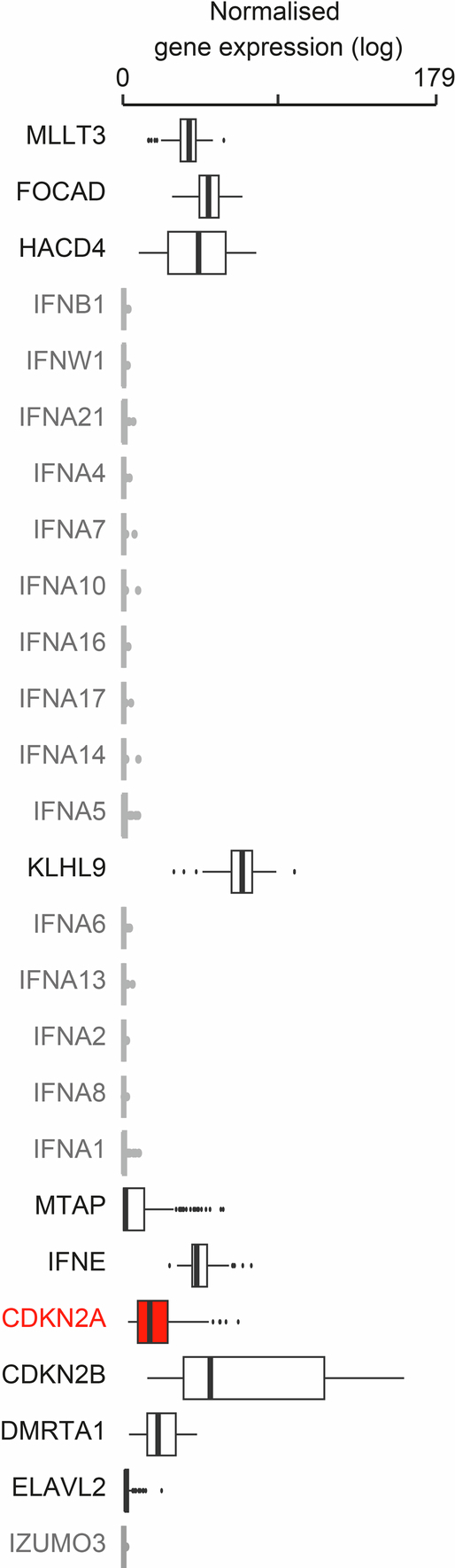
Expression of 9p21 genes in normal esophagus. Transcript Per Million (TPM) expression values of the 26 9p21 genes in esophagus samples from n = 139 healthy individuals. Data were derived from the Genotype-Tissue Expression (GTEx) repository (https://gtexportal.org/). Source data

We then investigated whether the co-occurring loss of other 9p21 genes could also contribute to poor survival, restricting the analysis to 779 EACs with WGS or WES data (Fig. 3e). Although *CDKN2A* was the most frequently occurring alteration in the locus, confirming that it is the event under positive selection, the other 25 genes were frequently co-lost with it (Fig. 3f). However, only ten 9p21 genes were expressed in EAC (Fig. 3g) or normal esophagus (Extended Data Fig. 3), suggesting that the loss of the remaining 16 genes likely had no functional consequences. We therefore tested the potential impact on survival of the ten 9p21 expressed genes by dividing patients with EAC in nine groups. Each of these groups represented at least 5% of the cohort and was composed of patients with the same 9p21 mutation and copy-number profile (Supplementary Table 5). Patients in all nine groups had worse survival than 413 patients with EAC with a wild-type 9p21 locus (FDR < 0.1; Fig. 3h and Supplementary Table 5). All patients lost *KLHL9*, *IFNE*, *MTAP*, *CDKN2A*, *CDKN2B* and *DMRTA1* (Fig. 3h), suggesting that alterations in these genes may contribute to poor prognosis.

### LoF of 9p21 genes has distinct consequences in BE and EAC

**Fig. 4:**
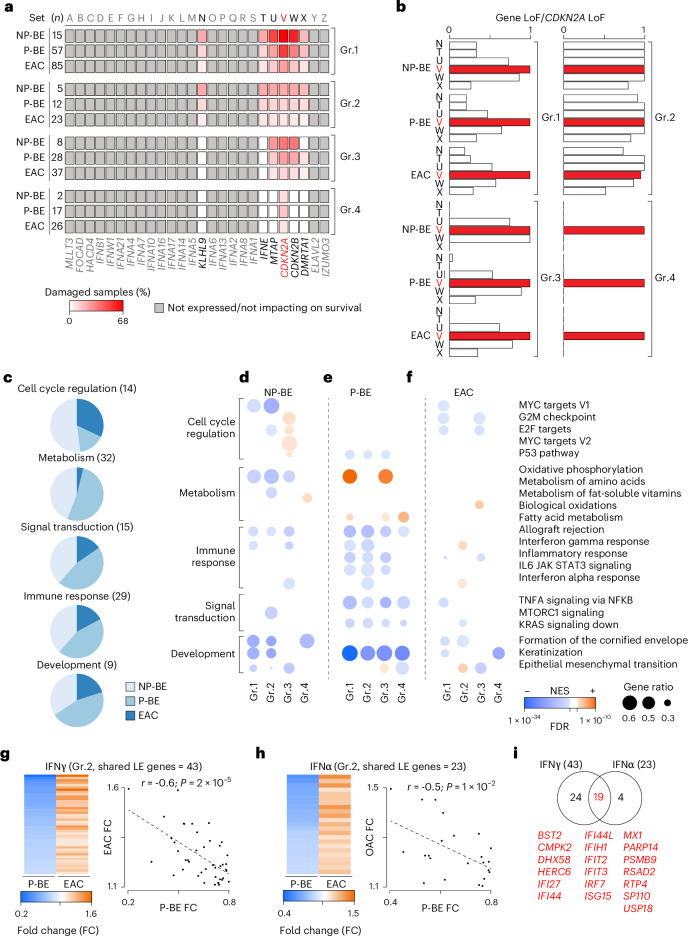
Functional consequences of 9p21 gene LoF in BE and EAC. **a**, Frequency of damaged 9p21 genes in the four groups estimated over *n* = 22 patients with NP-BE, *n* = 108 patients with P-BE and *n* = 337 patients with EAC with matched genomic and transcriptomic data. **b**, Proportions of samples with LoF in *KLHL9* (N), *IFNE* (T), *MTAP* (U), and *CDKN2B* (W) and *DMRTA1* (X) over samples with *CDKN2A* LoF (V) in each group of NP-BEs, P-BEs and EACs. The number of samples in each group and condition is reported. **c**, Relative proportion of dysregulated pathways in NP-BE, P-BE and EAC cohorts mapping to cell cycle regulation, metabolism, signal transduction, immune response and development. Numbers in brackets represent the number of unique pathways. **d**–**f**, Results of pre-ranked GSEA^48^ showing the normalized enrichment score (NES), FDR and gene ratio (number of leading-edge genes over the total expressed genes) of pathways dysregulated in each group of NP-BEs (**d**), P-BEs (**e**) and EACs (**f**). NES > 0 indicates pathway upregulation, whereas NES < 0 indicates downregulation. *P* values were estimated by permutation and corrected for multiple testing using the Benjamini–Hochberg method. **g**,**h**, Fold change of expression and correlation plot of the shared leading-edge (LE) genes of interferon gamma (**g**) and alpha (**h**) response pathways enriched in P-BE and EAC group 2 as compared to 9p21 wild-type samples. Coefficients and associated *P* values from two-sided Pearson’s correlation test are reported for both pathways. **i**, Overlap of leading-edge genes between interferon gamma and alpha response pathways enriched in P-BE and EAC group 2. The 19 shared genes are listed. FC, fold change. Source data

Our results suggested that the LoFs of *CDKN2A* and other 9p21 genes have functional and survival consequences that depend on time and context. Disentangling these variable effects is challenging because 9p21 genes are often co-damaged (Fig. 3f). To tease out the contribution of individual 9p21 genes, we divided 22 NP-BEs, 108 P-BEs and 337 EACs with matched genomic and transcriptomic data (Supplementary Table 1) into four groups (Fig. 4a). Each group had the same LoF profile of the six genes whose loss impacted survival (*KLHL9*, *IFNE*, *MTAP*, *CDKN2A*, *CDKN2B* and *DMRTA1*; Fig. 3h). Group 1 included all samples with *CDKN2A* LoF independently of the status of the other genes (Fig. 4b), closely resembling the cohorts tested in the survival analysis (Fig. 3a,d). The other three groups were subsets of group 1 with variable LoF frequency in the six genes (Fig. 4b).

We identified the dysregulated biological processes in each group as compared to the corresponding 9p21 wild-type samples by performing a pre-ranked gene set enrichment analysis (GSEA)^48^ in NP-BEs, P-BEs and EAC separately. Overall, we detected 72, 62 and 28 unique pathways significantly dysregulated (FDR ≤ 0.01) in NP-BE, P-BE and EAC, respectively (Supplementary Table 6). Almost 80% of these pathways mapped to only five biological processes, namely cell cycle regulation, metabolism, immune response, signal transduction, and development. Overall NP-BE and P-BE showed a higher fraction of dysregulated pathways than EAC (Fig. 4c), suggesting that 9p21 LoF had higher impact in premalignant conditions.

As expected, given *CDKN2A*, *CDKN2B* and *KLHL9* role in cell cycle regulation role, we found cell cycle dysregulation across groups and conditions except group 4 (*CDKN2A* LoF only; Fig. 4d–f and Supplementary Table 6), suggesting that the co-deletion of *KLHL9, CDKN2A* and *CDKN2B* maximizes the effect.

*CDKN2A* LoF alone might not be sufficient also to trigger metabolic or immune dysregulation (Fig. 4d–f and Supplementary Table 6). In this case *MTAP* and *IFNE* LoF could play a role given their functions in metabolic reprogramming^49,50^ and activation of immune response through metabolic regulation^51^, respectively. Interestingly, oxidative phosphorylation was consistently downregulated in NP-BE, upregulated in P-BE, and showed no difference in EAC (Fig. 4d–f and Supplementary Table 6). This once again suggested that the same genetic alterations may trigger different functional responses depending on the context. Similarly, the disruption of immune pathways differed between BE and EAC (Fig. 4d–f and Supplementary Table 6). Although interferon alpha and gamma responses were consistently downregulated in NP-BE and P-BE, both were upregulated in EAC, particularly in group 2 (Fig. 4b). Consistently, we observed a significant inverse correlation between expression fold changes of interferon gamma (Fig. 4g) and alpha (Fig. 4h) genes in BE and EAC groups 2 compared to 9p21 wild-type samples. Moreover, there was substantial overlap between altered genes in the two pathways (Fig. 4i), suggesting a comprehensive transcriptional reprogramming of interferon response. The most likely candidates for this reprogramming were again *MTAP*, given its recently reported ability to regulate the TME^11^, and *IFNE*, a type-1 interferon expressed in adult epithelia. Since the effect was most visible in group 2, which had LoF in both genes, and not in group 3, which had *MTAP* LoF and *IFNE* wild-type (Fig. 4a,b), the effect on interferon response might be due to *IFNE* loss.

*CDKN2A* LoF alone might instead be enough for the pervasive downregulation of keratinization genes given that these pathways were consistently dysregulated also in group 4 (Supplementary Table 6 and Fig. 4d–f).

### Loss of *IFNE* reduces immune infiltration in BE, but not in EAC

To further investigate the opposite effect of *IFNE* on interferon alpha and gamma response in BE and EAC (Fig. 4g,h), we quantified the infiltration of 18 immune cell populations in NP-BEs, P-BEs and EACs from their bulk transcriptomic data. We then compared the abundance of immune infiltrates between each of the four 9p21 LoF groups (Fig. 4a) and the corresponding 9p21 wild-type samples.

**Fig. 5:**
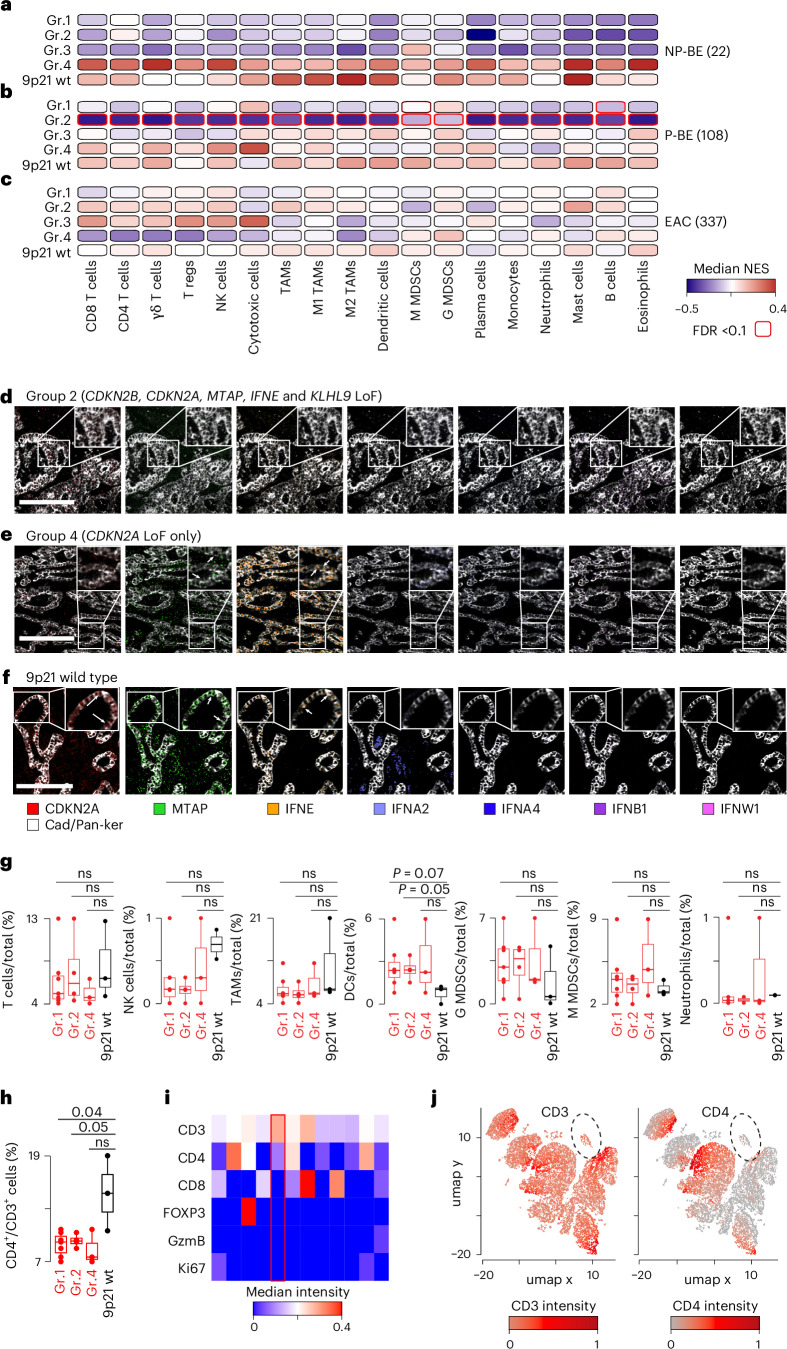
Impact of 9p21 gene loss on immune infiltration in BE and EAC. **a**–**c**, Comparison of NESs of 18 immune populations between 9p21 LoF and wild-type samples in *n* = 22 patients with NP-BE (**a**), *n* = 108 patients with P-BE (**b**) and *n* = 337 patients with EAC (**c**), respectively. NES distributions were compared using a two-sided Wilcoxon’s rank sum test and corrected for multiple testing using the Benjamini–Hochberg method. Numbers of samples are reported in brackets. Immune populations with significant differences (FDR < 0.1) are outlined in red. **d–f**, Representative IMC images from group 2 (*n* = 4 patients, **d**), group 4 (*n* = 3 patients, **e**) and 9p21 wild-type (*n* = 3 patients, **f**) EACs showing the expression of 9p21 targeted proteins and mRNAs. Cadherin-1 and pan-keratin denote tumor. Arrows indicate examples of epithelial staining. Scale bar: 200 μm. **g**, Relative abundance of immune cells over all cells in 9p21 LoF and wild-type EACs. Samples in groups 2 and 4 were pooled together to form group 1 (*n* = 7 patients). Distributions were compared using a two-sided Wilcoxon rank sum test. **h**, Relative abundance of CD4^+^ cells over all CD3^+^ cells in 9p21 LoF and wild-type EACs. Distributions were compared using a two-sided Wilcoxon rank sum test. **i**, Median marker intensity across the T cell clusters at a clustering resolution of 0.5. **j**, UMAP map of 9750 T cells in *n* = 10 patients with EAC. Cells were grouped in 12 clusters based on the expression of six markers and colored according to the mean intensities of CD3 and CD4. The cluster enriched in group 1 is circled. Boxplots in **g** and **h** show first and third quartiles, whiskers extend to the lowest and highest value within the 1.5X interquartile range and the line indicates the median. Samples in groups 2 (*n* = 4 patients) and 4 (*n* = 3 patients) were pooled together to form group 1 (*n* = 7 patients). For 9p21 wt groups *n* = 3 patients are shown for all populations, except NK and dendritic cells where samples with no staining were removed. DCs, dendritic cells; TAMs, tumour-associated macrophages. Source data

Immune infiltrates were depleted in NP-BE groups 1 to 3 (Fig. 5a and Supplementary Table 7) and P-BE groups 1 and 2 as compared to 9p21 wild-type samples (Fig. 5b and Supplementary Table 7), where the impact of *IFNE* LoF was more appreciable. This again suggested that the immune depletion is a consequence of *IFNE* loss consistent with recent observations of a cold TME when *IFNE*^10^ or the whole IFN locus^9^ are lost in melanoma ovarian, or pancreatic cancers (Supplementary Table 8). However, the same studies also reported an increased infiltration of T_reg_ cells, MDSCs and B cells (Supplementary Table 9) that we did not observe (Fig. 5a,b). The TME of group 4 (*CDKN2A* LoF only) was not significantly different to that of 9p21 wild-type samples in both NP-BE and P-BE, confirming that *CDKN2A* LoF does not directly interfere with the immune system.

Unlike other cancer types (Supplementary Table 8) and BE (Fig. 5a,b), we did not observe any significant TME difference between 9p21 LoF and wild-type EACs (Fig. 5c and Supplementary Table 7). To investigate this at higher resolution, we performed high-dimensional imaging mass cytometry (IMC) on tissue sections representative of group 1, group 2, group 4 and 9p21 wild-type EACs (Supplementary Table 9). We used a panel of 26 antibodies targeting structural, immune and 9p21-encoded proteins as well as RNAscope probes against *IFNE* and *IFNB1* mRNAs to increase the detection signal (Supplementary Table 10). We confirmed that group 2 lost the expression of all 9p21-encoded proteins in the tumor, whereas group 4 lost CDKN2A only compared to 9p21 wild-type EACs (Fig. 5d–f). Moreover, IFNE was the only interferon clearly expressed in EAC epithelium (Fig. 5d–f).

We performed single-cell segmentation of the IMC images to quantify T cells, NK cells, macrophages, dendritic cells, monocytic (M) and granulocytic (G) MDSCs, and neutrophils (Methods). We then compared the relative abundance of each immune population over all cells in each slide across EAC groups. We confirmed no significant difference in immune infiltration between 9p21 LoF and wild-type EACs, except for a borderline significant enrichment in dendritic cells in groups 1 and 2 (Fig. 5g). We further applied unsupervised clustering to T cells and macrophages, for which we had multiple markers (Supplementary Table 10), to test whether there was any difference in specific subpopulations. Again, we detected no major differences in any subpopulations of macrophages or T cells, except a borderline significant depletion of CD4^+^ T cells in groups 1 and 2 compared to 9p21 wild-type EAC (Fig. 5h–j). These results confirmed that, unlike BE, the loss of *IFNE* or any other 9p21 genes does not lead to any major difference in the TME of EAC.

### *CDKN2A* LoF favors squamous to columnar epithelium transition

**Fig. 6:**
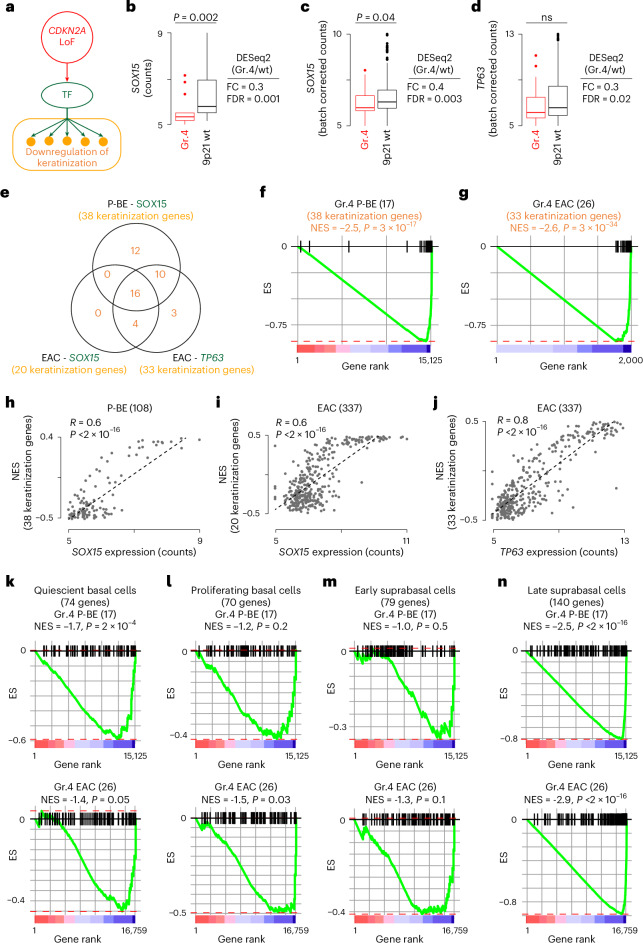
Impact of *CDKN2A* LoF on epithelium differentiation in P-BE and EAC. **a**, Gene regulatory network linking *CDKN2A* LoF to the downregulation of keratinization genes through TF deregulations. **b**–**d**, Distributions of gene expression values of *SOX15* in *n* = 17 patients with P-BE (*P* = 0.002) (**b**) and *SOX15* (*P* = 0.04) (**c**) and *TP63* (**d**) in *n* = 26 patients with EAC of group 4 and 9p21 wild type (31 P-BE and 184 patients with EAC, respectively). Distributions were compared using two-sided Wilcoxon’s rank sum test. FC and FDR from the differential gene expression analysis with DESeq2 (ref. ^77^) are also shown. Boxplot shows first and third quartiles, whiskers extend to the lowest and highest value within the 1.5× interquartile range and the line indicates the median. **e**, Overlap between keratinization genes targeted by *SOX15* and *TP63* in P-BE and EAC. **f**,**g**, Preranked GSEA plots using as signature keratinization genes targeted by *SOX15* in P-BE (**f**) and by *SOX15* and *TP63* in EAC (**g**). Genes were ranked from the most upregulated to the most downregulated in group 4 compared to 9p21 wild-type samples. For EAC, only the top 2,000 downregulated genes are shown. **h**–**j**, Correlation plots between keratinization GSEA NES and the gene expression values of *SOX15* in P-BE (**h**) and *SOX15* (**i**) and *TP63* (**j**) in EAC. Coefficients and associated *P* values from two-sided Spearman’s correlation test are reported. **k**–**n**, Preranked GSEA plots using gene signatures for quiescent basal cells (**k**), proliferating basal cells (**l**), early suprabasal cells (**m**) and late suprabasal cells (**n**) in P-BE and EAC group 4. *P* values in (**e–g** and **k–n**) were estimated by permutation. ES, enrichment score; TF, transcription factor; GSEA, gene set enrichment analysis. Source data

We observed a pervasive downregulation of processes responsible for terminal differentiation of keratinocytes, such as keratinization and formation of the cornified envelope, across all 9p21 LoF groups (Fig. 4d–f). In particular, P-BE and EAC groups 4 were associated with the downregulation of keratinization, suggesting that *CD2KNA* LoF alone was sufficient for triggering this process. To gain further mechanistic insights, we rebuilt the gene regulatory network linking *CD2KNA* LoF to keratinization in P-BE and EAC group 4 (Fig. 6a).

**Extended Data Fig. 4:**
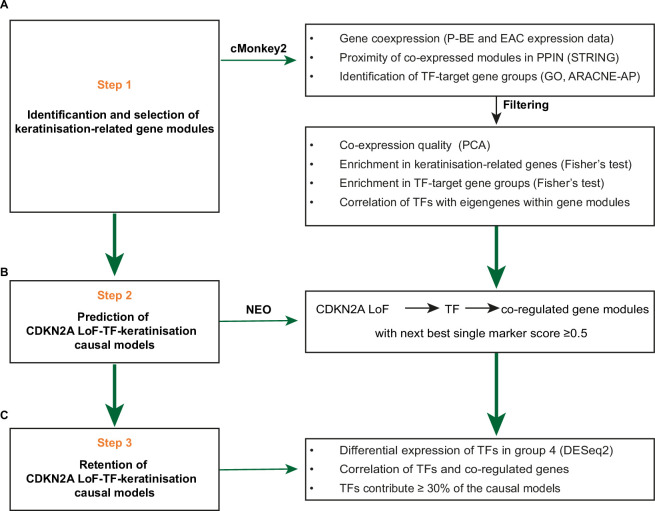
Workflow of causal gene network analysis. The workflow to infer the causal gene network linking *CDKN2A* LoF to the downregulation of keratinization was divided into three steps: **(A)** identification and filtering of the keratinization-related gene modules and associated transcription factors (TFs) using cMonkey2^88^ and ARACNE-AP^90^ (Step 1); **(B)** prediction of the causal models that link *CDKN2A* LoF to the dysregulation of keratinization genes through specific TFs using the network edge orienting (NEO) method^87,91^ (Step 2); and **(C)** retention of the causal models with differential expression (FDR < 0.1) of TFs in group 4 compared to 9p21 wild type samples assessed using DESeq2^77^ and significant positive correlation (R > 0.5 and FDR < 0.1) between TF expression and the GSEA NES score of the predicted targets in P-BEs and EACs (Step 3).

Using a three-step protocol (Extended Data Fig. 4a–c and Methods), we identified 8 and 14 causal models in P-BE and EAC, respectively, linking *CDKN2A* LoF directly to keratinization gene downregulation through the perturbation of two TFs (SOX15 and TP63; Supplementary Table 11). We further confirmed that these TFs were significantly downregulated in P-BE (Fig. 6b) and EAC (Fig. 6c,d) groups 4 as compared to 9p21 wild-type samples. Overall, the gene modules controlled by SOX15 and TP63 included 45 keratinization genes (Supplementary Table 11), 16 (36%) of which were shared across all gene modules and 30 were shared between SOX15 and TP63 (Fig. 6e). Therefore, the downregulation of these two TFs in *CDKN2A* LoF samples led to a comprehensive downregulation of the keratinization transcriptional program, as confirmed by a pre-ranked GSEA^48^ using keratinization gene-derived signatures in P-BE (Fig. 6f) and EAC (Fig. 6g). Moreover, *SOX15* and *TP63* gene expressions were positively correlated with the enrichment score of the keratinization genes (Fig. 6h–j), again confirming that the two TFs control their expression.

SOX15 regulates transcription of a large number of genes specific to esophageal epithelium^52^, and TP63 is essential for development and maintenance of all stratified epithelia^53^. The transition from esophageal squamous epithelium to intestinal columnar epithelium is a key feature in the initiation of BE and EAC^54^. Our data suggest that *CDKN2A* LoF leads to a downregulation of the transcriptional program responsible for the maintenance of the squamous epithelium more robust and persistent than in *CDKN2A* wild-type samples. Although this did not prove a direct causative role of *CDKN2A* LoF, it shows correlation between the two events. To further test the link between *CDKN2A* LoF and suppression of squamous epithelium, we performed preranked GSEA^48^ using four independent gene signatures characteristic of cells composing the esophageal epithelium, namely quiescent basal cells, proliferating basal cells, early suprabasal cells and late suprabasal cells^55^. We observed global downregulation of all four signatures in EAC and quiescent basal cells and late suprabasal cells in P-BE (Fig. 6k–n). These results supported our hypothesis that *CDKN2A* LoF exacerbates a phenotype typical of EAC and that this may contribute to more aggressive tumors.

## Discussion

In this study, we dissected the role of *CDKN2A* and other 9p21 genes in EAC evolution, from the transformation of premalignant BE to the impact on patient survival.

Despite being an EAC driver, the early loss of *CDKN2A* has a tumor-suppressive role supported by its higher occurrence in NP-BE than P-BE and EAC. This is consistent with other drivers whose alterations are more frequent in normal tissues than cancer, including *ERBB2*, *ERBB3*, *KRAS* and *NOTCH1* (ref. ^56^). The anti-tumorigenic function of *NOTCH1* is exerted through an increased fitness of *NOTCH1* mutant cells that outcompete early tumors^57^. For *CDKN2A* we propose a different mechanism whereby *TP53* mutations reduce the proliferative capacity of *CDKN2A* mutant BE cells that are therefore counter-selected. As *TP53* loss is a strong driver of EAC initiation, the decrease of its occurrence induced by *CDKN2A* LoF also decreases tumor initiation. Recent studies observed tumor formation upon induction of *TP53* and *CDKN2A* double KO in mouse or human gastroesophageal organoids^58–60^. However, in these studies, *TP53* and *CDKN2A* inactivation was induced concomitantly, that is targeting both genes at the same time. However, in real precancer conditions, such as BE, mutations are acquired over time and cells with different genetic makeup and fitness coexist and compete for nutrient and space. Our results confirm that the order of mutations is key to decide the fate of mutant cells in the initial phases of tumor evolution^56^.

It is tempting to speculate that the tumor-preventive role of early *CDKN2A* LoF could be further developed as a marker of favorable prognosis in nondysplastic BE. Endoscopic surveillance of BE is an integral component of the current EAC prevention paradigm, but the rate of progression to EAC is only 0.54/100 patient-years^61^. Identifying BE cases with a lower risk of progression could substantially improve patient management, decreasing the burden of endoscopy for patients who have low chances to develop cancer.

*CDKN2A* LoF is the most frequent event in 9p21 locus, implying that the co-occurring loss of other 9p21 genes is due to genetic hitchhiking, with variable effects on cell cycle, oxidative phosphorylation, and interferon response depending on the stage and context of BE and EAC evolution. Most notably, *IFNE* exerts a tumor-suppressive role in BE, but not in EAC, by reducing IFN response and inducing a cold immune microenvironment. Despite several reports of a lower infiltration of immune cells in cancers with reduced *CDKN2A* expression^62,63^, *CDKN2A* LoF alone does not change the immune composition of BE or EAC TME. This may be due to tumor-specific effects or to the fact that at least some cancer-promoting roles previously attributed to *CDKN2A* LoF are in fact triggered by the loss of other 9p21 genes.

The association of *CDKN2A* LoF with bad prognosis is also context dependent and detectable only in patients with EAC. It appears unrelated to the role of *CDKN2A* in cell cycle since alterations in other cell cycle regulators can drive EAC without affecting survival. A contribution towards a more aggressive EAC phenotype is likely due to a combination of effects, including the pervasive suppression of transcriptional programs responsible for the maintenance of squamous epithelium. Although this is a common feature of BE and EAC^54^, it is significantly more pronounced when *CDKN2A* is lost and is achieved through *TP63* and *SOX15* downregulation. This could be an indirect effect of *CDKN2A* LoF on the E2F transcriptional program, as iASPP, which controls *TP63* expression^64^, is a target of E2F1 (ref. ^65^) and *SOX15*, in turn, is a target of TP63 (ref. ^66^).

Our study introduces the intriguing concept that the functional consequences of alterations in cancer genes may change during the evolution of disease, from preventing cancer transformation in the premalignant setting to favoring a more aggressive disease at later stages. This fits the emerging scenario whereby the functional consequences of cancer alterations and the fitness provided to the mutant cell are not invariable but depend on the cell genetic background^67^, neighborhood^57^ or order of events as we showed here. If proven of general applicability, this may lead to a paradigm shift with consequences on the understanding and treatment of cancer.

## Methods

### Ethical approval

Written consent was obtained from all patients with BE or EAC from the University of Cambridge (UoC) whose samples were sequenced for this study (REC: 10/H0305/1 & IRAS:15757). Samples were collected at endoscopy, staging laparoscopy, endoscopic mucosal resection or surgical resection and then snap frozen in liquid nitrogen. Samples were then embedded in optimal cutting temperature media for cutting of 1 × 3 µM slide to be H&E stained and reviewed by a pathologist. Only tumor samples of >50% cellularity and BE samples with high intestinal metaplasia content proceeded to sequencing.

### Sample collection

Single-nucleotide variants (SNVs), indels and copy-number data for 1,032 primary EACs were collected from published studies and de novo sequenced samples (Supplementary Table 1). In particular, WGS from 706 EACs was performed at UoC (EGAD00001011191 and EGAD00001006083, https://ega-archive.org/). WES data for 73 TCGA EACs were downloaded from the Genomic Data Commons portal (https://portal.gdc.cancer.gov/). Damaged genes for 253 Memorial Sloan Kettering Cancer Center (MSKCC) EACs that underwent targeted re-sequencing of 528 (ref. ^37^), 477 (ref. ^6^) and 970 (ref. ^38^) genes were downloaded from the cBioPortal (https://www.cbioportal.org/). In cases of multiple samples per patient, the sample with *CDKN2A* LoF was retained. Clinical data for the TCGA and MSKCC cohorts were obtained from the same sources. For the UoC cohort, clinical data were derived from LabKey (https://occams.cs.ox.ac.uk/labkey). Bulk RNA-seq data were available for 337 EACs, all of which had matched WGS or WES (Supplementary Table 1). Of these, 264 were sequenced at the UoC (EGAD00001011190) and 73 were derived from TCGA. Methylation data were available for 256 EACs (EGAD00010001822 (ref. ^40^) and TCGA^32^; Supplementary Table 1).

WGS, WES and clinical data for 356 BEs were obtained from UoC (EGAD00001011191 and EGAD00001011189, which also includes samples from Katz-Summercorn et al.^43^ and Killcoyne et al.^44^) and from the Fred Hutchinson Cancer Research Center (FHCRC)^15,42^ (Supplementary Table 1). As for EAC, in cases of multiple samples per patient, the sample with *CDKN2A* LoF was retained. BE cases were classified as progressors (P-BE, 257) or non-progressors (NP-BE, 99) based on whether patients progressed or not to high-grade dysplasia or EAC in a follow-up period of up to 17 years (Supplementary Table 1).

Paired WGS BE and EAC data were available for 86 cases (EGAD00001011191 and EGAD00001006083, which also include samples from Noorani et al.^34^, Ross-Innes et al.^35^and Katz-Summercorn et al.^43^). Methylation data for 57 BE cases were derived from UoC (EGAD00010001838 (ref. ^40^) and EGAD00010001972 (ref. ^43^)). Bulk RNA-seq data for 108 P-BEs and 22 NP-BEs were sequenced at the UoC (EGAD00001011190, including samples from Katz-Summercorn et al.^43^) (Supplementary Table 1).

### DNA and RNA extraction, library preparation and variant calling

DNA and RNA were extracted using Qiagen AllPrep Mini kits, using a Precellys for tissue dissociation after all excess OCT was removed. Extracted nucleic acids were quantified by Qubit. Libraries were then prepared using Illumina PCR Free methods and sequenced on HiSeq 4000 or NovaSeq platforms. Paired-end whole-genome sequencing at 50× target depth for EACs, P-BEs and NP-BEs and 30× target depth for matched normal (blood) was performed by Illumina, the Sanger Institute, or the CRUK Cambridge Institute on Illumina platforms. Quality checks were performed using FastQC (http://www.bioinformatics.babraham.ac.uk/projects/fastqc/). For mutation calling, sequencing reads were aligned against the reference genome (hg19/GRCh37) using BWA-MEM^68^. Aligned reads were then sorted into genome coordinate order and duplicate reads were flagged using Picard MarkDuplicates (http://broadinstitute.github.io/picard). Strelka^69^ 2.0.15 was used for calling single nucleotide variants and indels. Sample purity and ploidy values were estimated using ASCAT-NGS 2.1^70^. Copy-number alterations (CNAs) after correction for estimated normal-cell contamination were inferred using ASCAT from read counts at germline heterozygous positions estimated by GATK 3.2-2 HaplotypeCaller^71^. Shallow WGS data for 75 BE cases^44^ were processed with the QDNAseq package using 50-kb bins including GC-bias correction, segmentation and generation of copy-number calls and used to identify homozygously deleted and amplified genes. Because the read depth was only 0.4×, mutation calls could not be performed.

### Annotation of damaged genes and EAC drivers and clonality analysis

For WGS (UoC, FHCRC) and WES (TCGA) data, SNV, indel and copy-number calls were taken from the original publications or derived as described above. ANNOVAR^72^ (April 2018) and dbNSFP^73^ v3. 0 were used to annotate the effect of mutations and indels. Only SNVs and indels with damaging effects on the proteins as previously described^1^ were further retained. Briefly, these included (1) truncating (stopgain, stoploss, frameshift) mutations; (2) missense mutations predicted by at least seven methods^1^.

CNA segments from ASCAT were intersected with the exonic coordinates of 19,641 unique human genes^1^, and a gene was considered amplified, homozygously or heterozygously deleted if at least 25% of its length overlapped with an amplified (CNA > twice sample ploidy) or homozygously (CNA = 0) or heterozygously deleted (CNA = 1) segment, respectively. Genes with at least one damaging SNV or indel as well as amplified and homozygously deleted genes were considered damaged. Genes with heterozygous deletion of one allele and at least a damaging SNV or indel in the other (double hit), were also considered damaged. Genes with only heterozygous deletions were not considered damaged. For *CDKN2A* only, *CDKN2A* silencing via methylation was also considered. Raw methylation data were processed with the minfi package and normalized with the BETA mixture model BMIQ of the ChAMP package. *CDKN2A* was considered epigenetically silenced if the cg12840719 probe located within 1,500 bp from its transcription start site^40^ had a methylation β value ≥ 0.3 and its *CDKN2A* value was comparable to samples with homozygously deleted CDKN2A. The distribution of damaged genes across EAC and BE cohorts is shown in Extended Data Fig. 1. Mutated, amplified and homozygously deleted genes for the MSKCC cohort^6,37,38^ were downloaded from the cBioPortal.

Five hundred eighty out of 779 EACs with WGS or WES data (Supplementary Table 1) had damaging alterations in *TP53* or *CDKN2A* and were further analyzed to measure mutation clonality as described previously^74^. Briefly, the probability of each damaging mutation to have a cancer cell fraction (CCF) from 0.01 to 1 incremented by 0.01 was calculated given the observed variant allele frequency (VAF), gene copy-number status in the cancer and normal sample and sample purity. Then, the clonal probability of a *TP53* or *CDKN2A* mutation was calculated as the cumulative probability of CCF being >0.95. A damaging mutation was considered clonal if its clonal probability was >50%.

A list of 40 EAC canonical drivers was obtained from the Network of Cancer Genes (NCG7.1, http://www.network-cancer-genes.org)^1^. Additionally, 34 EAC drivers that undergo CNA were collected through manual curation of the literature. Only 54 of the resulting 74 EAC drivers were present also in the gene panel used in the MSKCC studies and these were considered for further analysis (Supplementary Table 2).

### Cell lines and gene expression quantification

In vitro experiments were carried out using the CP-A (KR-42421) BE cells from the Francis Crick Institute cell service facility (ATCC catalog number CRL-4027). Cells were grown at 37 °C and 5% CO_2_ in keratinocyte serum-free medium supplemented with 50 µg ml^−1^ bovine pituitary extract and 5 ng/ml recombinant human EGF (Thermo Fisher). Total RNA was extracted from CP-A wild-type cells and *TP53* KO clones using the Direct-zol RNA miniprep kit (ZymoResearch) and reverse transcribed using the High-capacity cDNA reverse transcription kit (Thermo Fisher). Predesigned Taqman gene expression assays for *CDKN2A* and *TP53* were used (Life Technologies; Supplementary Table 4), whereas gene-specific primers and probe were designed for *ACTB* (Merck; Supplementary Table 4). Real-time quantitative PCR (rt-qPCR) was performed in duplicate using QuantiTect probe PCR mastermix (Qiagen) and repeated three times. Gene relative expression was calculated using the 2^−ΔΔCt^ method and *ACTB* as endogenous control. A pool of human RNA was used as a positive control.

### *TP53* gene editing and cell proliferation assay

To induce *TP53* KO via CRISPR-Cas9 gene editing, 3.5 × 10^5^ CP-A cells were co-transfected with two *TP53*-specific gRNAs (Supplementary Table 4) and Alt-R S.p.Cas9-Nuclease V3 (IDT) by nucleofection using the P3 Primary Cell 4D-NucleofectorTM X Kit S (Lonza) on a 4D-Nucleofector (Lonza). After nucleofection, single cells were plated in individual wells to form clonal colonies. Genomic DNA of nucleofected colonies was extracted using PureLink Genomic DNA mini kit (Invitrogen) and regions surrounding the targeted sites were amplified from genomic DNA of nucleofected colonies using HotStartTaq Plus DNA polymerase (Qiagen) and primers including Illumina adapters (Supplementary Table 4). Amplicons were sequenced on Illumina Novaseq using the paired-end protocol to confirm editing (BAM files: 10.5281/zenodo.12918301).

Cell proliferation of *TP53* KO and wild-type CP-A cells was measured every 24 h for 3 days, starting 3 h after seeding the cells using CellTiter-Glo Luminescent Cell Viability Assay (Promega). Briefly, 2 × 10^3^ cells per well were seeded on 96-well plates in a final volume of 100 μl per well. At each time point, 100 μl of the CellTiter-Glo reagent was added to the wells and luminescence was measured after 30 minutes using the Infinite F200 Pro plate reader (Tecan). For all proliferation assays, two or four technical replicates per condition were measured at each time point and each measure was normalized to the average time zero measure for each condition. Each experiment was repeated three independent times. Conditions were compared using the two-sided Student’s *t*-test.

### Logistic regression and survival analysis

Logistic regression with Firth bias correction^75^ was used to test the difference between two models of EAC initiation in the entire BE (P-BE and NP-BE) cohort. The first model assumed *TP53* LoF as the only driver (model 1), whereas the second model assumed that both *TP53* and *CDKN2A* LoF impacted on EAC initiation (model 2). The models were developed using the package logistf v1.25.0 and compared using the anova function in R. The two models were used to estimate the numbers of expected BE cases that progressed to EAC according to corresponding genomic status of *TP53* and *CDKN2A*. The β coefficients for *TP53* and *CDKN2A* LoF were obtained from the regression models and the p-values were calculated using the chi-squared test. Negative or positive β coefficient values indicated cancer-protective or cancer-promoting roles, respectively. The β coefficient (β) of *CDKN2A* LoF in model 2 was used to estimate the odds of progression as:

$${odds}={e}^{{{\beta }}}$$

The results of the whole analysis are reported in Supplementary Table 3.

Kaplan-Meier survival analysis was performed with survminer v.0.4.9 using the log-rank method. The analysis of the survival effect of *CDKN2A* co-damage with other 9p21 genes was performed only on 779 patients with EAC with WGS or WES data as the information on the genomic alteration of all 9p21 genes was not available in the targeted re-sequencing studies. Log-rank method was used to estimate *P* values, which were then corrected for multiple hypothesis testing using the Benjamini–Hochberg method, when needed.

### RNA-seq, gene set enrichment and immune infiltration

Paired-end RNA-seq for EAC, P-BE and NP-BE from UoC was performed at the CRUK Cambridge Institute on Illumina platforms and quality checks were performed using FastQC. Reads were aligned using STAR with ENSEMBL gene annotation. Reads per gene were quantified using the summariseOverlaps function from the GenomicRanges package. Raw read counts of 18,846 human genes shared between the UoC and TCGA cohorts were extracted from the corresponding BE and EAC RNA-seq datasets. SMIXnorm v0.0.0.9 (ref. ^76^) was used to estimate the probability of expression of these genes across all samples. Genes with a probability of expression below 0.9 were filtered out, resulting in 16,901 retained genes in EAC, 15,134 in P-BE and 15,866 in NP-BE, respectively.

Twenty-two NP-BEs, 108 P-BEs and 337 EACs with matched genomic and transcriptomic data (Supplementary Table 1) were divided into four groups depending on the mutation and copy-number profiles of the six 9p21 genes (*KLHL9*, *IFNE*, *MTAP*, *CDKN2A*, *CDKN2B* and *DMRTA1*) with impact on survival. Differential gene expression analysis was performed between each of these groups and the corresponding 9p21 wild-type EACs (184), P-BEs (31) and NP-BEs (6) using DESeq2 v1.38.3 (ref. ^77^) after correction for the batch effect with DESeqDataSetFromMatrix. Genes were ordered according to log2 fold-change values and used for preranked GSEA using fgsea v1.24.0 (ref. ^48^) against 50 gene sets from MSigDB v7.5.1 (ref. ^78^) and 1,303 level 2-8 pathways from Reactome v.72 (ref. ^79^) containing between 10 and 500 expressed genes and excluding the disease hierarchical level. The resulting *P* values were corrected for multiple testing in each analysis separately using the Benjamini–Hochberg method. Pathway redundancy was removed accounting for the extent of overlap between leading-edge genes; that is, the genes that contributed the most to the enrichment. If the number of unique leading-edge genes in a pathway was higher than the shared and the unique leading-edge genes in the other pathway, the latter was removed. If the number of shared leading-edge genes between two pathways was higher than the unique leading-edge genes in both, the pathway with the higher FDR was removed. Retained processes are reported in Supplementary Table 6.

To estimate the abundance of immune cell populations from bulk RNA-seq data, raw read counts of the expressed genes from 22 NP-BEs, 108 P-BEs and 337 EACs were normalized to transcripts per million values after batch correction with ComBat-seq^80^. Resulting transcripts per million were used as input for ConsensusTME v0.0.1 (ref. ^81^) as implemented in immunedeconv v2.1.0 to estimate the NES using 16 esophageal carcinoma immune signatures. To further estimate the abundance of MDSCs, two M-MDSC and G-MDSC signatures^82^ were used in ConsensusTME custom mode.

### RNAScope and imaging mass cytometry

A panel of 26 antibodies targeting structural markers, immune markers, three 9p21 proteins and three RNAScope probes against *IFNE*, *IFNB1* and *PPIB* mRNAs was assembled (Supplementary Table 10). RNAScope staining was detected using metal-tagged antibodies as previously described^83^. Sixteen of these antibodies were already metal-tagged (Standard Biotools), whereas eleven were carrier-free and tagged using the Maxpar X8 metal conjugation kit (Standard Biotools). The whole panel was tested in EAC FFPE sections using three dilutions ranging from 1:100 to 1:3,500 and the dilution giving the highest signal-to-noise ratio was chosen for each antibody (Supplementary Table 10).

Five-micrometer-thick sections were obtained from FFPE blocks of ten patients with EAC selected based on their 9p21 gene profile (Supplementary Table 9). Slides were incubated for 1 h at 60 °C, loaded on a Leica Bond autostainer (Leica Biosystems) and processed using the RNASCope LS Multiplex Fluorescent Assay following manufacturer’s instructions and *IFNE*, *IFNB1* and *PPIB* probes at a 1:50 dilution. C2 oligos were developed with TSA-digoxinenin, C3 oligos with TSA-biotin and C1 oligos with TSA-FITC (diluted 1:200 in TSA buffer). Slides were blocked for 2 h at room temperature in a Sequenza rack (Thermo Fisher Scientific). Slides were incubated overnight at 4 °C with the mix of metal-conjugated antibodies, washed, and incubated with the DNA intercalator Cell-ID Intercalator-Ir (Standard Biotools). Slides were removed from the Sequenza rack, air-dried and loaded into the Hyperion Imaging System (Standard Biotools). Regions of interest were manually selected to contain areas with tumor and immune cells by a certified pathologist (M.R.J). Regions of about 1.44 mm^2^ were laser-ablated within the preselected regions of interest at 1 μm pixel^−1^ resolution and 400 Hz frequency.

IMC image analysis was performed using SIMPLI^84^. TIFF images for each metal-tagged antibody and DNA intercalator were obtained from the raw.txt files of the ablated regions. Pixel intensities for each channel were normalized to the 99th percentile of the intensity distribution. Background pixels of the normalized images were removed with CellProfiler4 (ref. ^85^) using global thresholding and processed images were verified by an expert histologist (J.S.). Single-cell segmentation was performed using CellProfiler4 (ref. ^85^) to identify cell nucleus (DNA1 channel) and membrane (cadherin-1, pan-keratin, CD3, CD8, CD4, CD11b, CD11c, NCAM1, CD68, CD27, CD163, CD16, CD15 and CD14). Obtained cells were phenotyped based on at least 10% overlap with the masks of individual cell types in the following order: (1) CD15^+^ and CD16^+^ for neutrophils; (2) NCAM1^+^ for NK cells; (3) CD11c^+^ for dendritic cells; (4) CD68^+^ for macrophages; (5) CD14^+^ for M-MDSCs; (6) CD15^+^ for G-MDSCs; (7) CD3^+^ for T cells; (8) cadherin-1 and pan-keratin for tumor cells and (9) vimentin for stromal cells. Cells with <10% overlap with any mask were left unassigned.

Unsupervised clustering was performed separately on CD3^+^ T cells and CD68^+^ macrophages using Seurat v.2.4 (ref. ^86^), with random seed = 123 and 0.3, 0.5, 0.7 and 0.9 cluster resolutions. Markers used for clustering were CD3, CD4, CD8, FOXP3, GzMB and Ki67 for T cells, and CD68, CD11c, HLA-DR/DP/DQ and CD163, CD11b and Ki67 for macrophages. Silhouette score of each cluster was calculated using v.2.1.6 package. The resolution with the highest median silhouette score was identified as the best clustering resolution for each cell type.

### Keratinization causal regulatory network analysis

Causal networks linking *CDKN2A* LoF to the downregulation of keratinization were inferred using a three-step protocol modified from^87^, separately for P-BE and EAC (Extended Data Fig. 4a–c). In the first step, co-regulated gene modules were identified using cMonkey2 (ref. ^88^) based on gene co-expression, proximity in the protein-protein interaction network (PPIN) and enrichment in transcription factor (TF) targets. Co-expressed genes were identified from the top 50% most variably expressed genes in P-BE and EAC after converting read counts into *z*-scores using DESeq2 v1.38.3 (ref. ^77^). Proximity in the PPIN was measured using the human weighted PPIN from STRING v11.5 (ref. ^89^). GO:0006355 term of Gene Ontology (release 2022-05) was used to identify 1,471 TFs. These were in turn used as input for ARACNE-AP^90^ together with P-BE and EAC gene expression data to identify TF-target pairs. cMonkey2 was run with a fixed number of iterations (*n* = 2,000) and seed value (*n* = 123) for the initialization step to ensure reproducibility. The number of gene modules (k) was determined as:

$$k=\frac{{nAG}* {nBpG}}{{nGpB}}$$

where *nAG* was the number of analyzed genes, *nBpG* was the maximum number of gene modules each gene could appear in (fixed to 2), and *nGpB* was the average number of genes per gene module (fixed to 30). Identified gene modules were then filtered based on (i) co-expression quality according to the first principal component (FDR ≤ 0.1 and variance explained ≥0.32 for P-BE and ≥0.25 for EAC), (ii) functional enrichment in keratinization-related genes (two-sided Fisher’s test *P* ≤ 0.01), (iii) enrichment in TF target genes (two-sided Fisher’s test *P* value ≤ 0.01), and (iv) correlation of TFs with gene module eigengenes, that is genes that explain the maximum expression variance. In the second step, the single.marker.analysis function of the Network Edge Orienting^87^ method was used to infer causal models where *CDKN2A* LoF causally affected the expression of specific TFs, which, in turn, altered keratinization gene modules. To assess statistical significance, the next best single marker score was defined as the log10 probability of the causal model divided by the log10 probability of the next best fitting alternative model^91^ and causal models with next best single marker score ≥0.5 were considered significant. In the third step, significant causal models were further retained if (1) TFs were differentially expressed (FDR < 0.1) in group 4 as compared to 9p21 wild-type P-BEs and EACs and (2) there was significant positive correlation (*R* > 0.5 and FDR < 0.1) between TF expression and the GSEA NES score of the predicted targets in P-BEs and EACs. Finally, only TFs contributing to ≥ 30% of the significant causal models were retained. The final list of significant causal models and associated TFs is reported in Supplementary Table 11.

### Statistical analysis and reproducibility

All statistical tests were performed in R v.4.3.1 and results were plotted using ggplot2 v.3.4.4 and ggpubr v.0.6.0. All distributions were compared using two-sided Wilcoxon rank-sum test. Growth curves were compared using two-sided Student’s *t*-test. Two-sided Fisher’s exact test was used to compare categorical variables. Kaplan–Meier analysis with a log-rank test was performed for survival analysis. *P* value estimation for pre-ranked GSEA was based on an adaptive multilevel split Monte-Carlo scheme. Pearson’s correlation test and Spearman’s rank correlation test were used to assess correlation significance. Benjamini–Hochberg method was used to account for multiple testing when needed and false discovery rate <0.1 was considered as significant. No statistical method was used to predetermine sample size, as sample sizes were as large as possible considering available data. No data were excluded from any analysis. Data normalization was performed before analysis, but this was not formally tested, Experiments were not randomized, and the investigators were not blinded to allocation during experiments and outcome assessment. To ensure results reproducibility, all experiments were conducted in replicates as specified in the corresponding methods. Further information on research design is available in the Nature Research Reporting Summary linked to this article.

### Reporting summary

Further information on research design is available in the Nature Portfolio Reporting Summary linked to this article.

## Supplementary information

Reporting Summary

Supplementary Tables 1–11 Supplementary Table 1: Samples used in the study. Supplementary Table 2: Curated list of EAC canonical drivers. Supplementary Table 3: Results of logistic regression analysis. Supplementary Table 4: Oligos used in the study. Supplementary Table 5: Survival analysis of patients with 9p21 co-damaged genes. Supplementary Table 6: Dysregulated pathways in 9p21 LoF samples. Supplementary Table 7: Immune infiltration in 9p21 LoF and wild-type samples. Supplementary Table 8: Literature support for the effect of 9p21 gene LoF on immune infiltration. Supplementary Table 9: EACs used for RNAScope-Imaging mass cytometry. Supplementary Table 10: Markers used for RNAScope-Imaging mass cytometry. Supplementary Table 11: Causal models associated with decreased keratinization.

## Source data

Source Data Fig. 1 Numerical source data.

Source Data Fig. 2 Numerical source data.

Source Data Fig. 3 Numerical source data.

Source Data Fig. 4 Numerical source data.

Source Data Fig. 5 Numerical source data.

Source Data Fig. 5 IMC images.

Source Data Fig. 6 Numerical source data.

Source Data Extended Data Fig. 1 Numerical source data.

Source Data Extended Data Fig. 2 Numerical source data.

Source Data Extended Data Fig. 3 Numerical source data.

## Data Availability

DNA and RNA sequence data for the UoC cohort were deposited at the European Genome-phenome Archive with the following accession IDs: WGS (EGAD00001011191, EGAD00001006083), shallow WGS (EGAD00001011189), bulk RNA sequencing (EGAD00001011190). WES for 73 TCGA EACs were downloaded from the Genomic Data Commons portal (https://portal.gdc.cancer.gov/). Mutated genes for 253 Memorial Sloan Kettering Cancer Center (MSKCC) EACs that underwent targeted re-sequencing were downloaded from the cBioPortal (https://www.cbioportal.org/). Methylation data for EACs were derived from UoC (EGAD00010001822) and TCGA (https://portal.gdc.cancer.gov/). Methylation data for BE were derived from UoC (EGAD00010001838 and EGAD00010001972). BAM files of wild-type and *TP53* edited CP-A cells were deposited at Zenodo (10.5281/zenodo.12918301) (ref. ^92^). UoC WGS, sWGS, RNA-seq and methylation data of the human patients are under controlled access by ICGC (International Cancer Genome Consortium) due to privacy and security protection of personal data. The reasons and conditions for controlled access are described here (https://www.icgc-argo.org/page/132/data-access-and-data-use-policies-and-guidelines). The data can be accessed via the ICGC portal upon request to the ICGC Data Access Compliance Office here: https://docs.icgc-argo.org/docs/data-access/daco/applying. Source data for Figs. 1–6 and Extended Data Figs. 1–3 have been provided as Source Data files. All other data supporting the findings of this study are available from the corresponding author on reasonable request. Source data are provided with this paper.

## Code availability

No unique or custom code was developed for this study.
