# Supplementary material for: Context-dependent effects of CDKN2A and other 9p21 gene losses during the evolution of esophageal cancer
Source: Nat Cancer. 2025 Jan 3;6(1):158–74. doi: 10.1038/s43018-024-00876-0 (PMC11779637; doi:10.1038/s43018-024-00876-0)
Supplement: Supplementary file 1 — Reporting Summary [file 43018_2024_876_MOESM1_ESM.pdf]

nature portfolio

Corresponding author(s): Francesca D. Ciccarelli

Last updated by author(s): Aug 2, 2024

Reporting Summary

Nature Portfolio wishes to improve the reproducibility of the work that we publish. This form provides structure for consistency and transparency in reporting. For further information on Nature Portfolio policies, see our Editorial Policies and the Editorial Policy Checklist.

Statistics

For all statistical analyses, confirm that the following items are present in the figure legend, table legend, main text, or Methods section.

n/a	Confirmed	
☐	☒	The exact sample size (*n*) for each experimental group/condition, given as a discrete number and unit of measurement
☒	☐	A statement on whether measurements were taken from distinct samples or whether the same sample was measured repeatedly
☐	☒	The statistical test(s) used AND whether they are one- or two-sided<br>*Only common tests should be described solely by name; describe more complex techniques in the Methods section.*
☒	☐	A description of all covariates tested
☐	☒	A description of any assumptions or corrections, such as tests of normality and adjustment for multiple comparisons
☐	☒	A full description of the statistical parameters including central tendency (e.g. means) or other basic estimates (e.g. regression coefficient) AND variation (e.g. standard deviation) or associated estimates of uncertainty (e.g. confidence intervals)
☐	☒	For null hypothesis testing, the test statistic (e.g. *F*, *t*, *r*) with confidence intervals, effect sizes, degrees of freedom and *P* value noted<br>*Give P values as exact values whenever suitable.*
☒	☐	For Bayesian analysis, information on the choice of priors and Markov chain Monte Carlo settings
☒	☐	For hierarchical and complex designs, identification of the appropriate level for tests and full reporting of outcomes
☐	☒	Estimates of effect sizes (e.g. Cohen's *d*, Pearson's *r*), indicating how they were calculated

Our web collection on statistics for biologists contains articles on many of the points above.

Software and code

Policy information about availability of computer code

Data collection	No software used for data collection.
Data analysis	Softwares and packages used for the data analysis and representation are as follows:<br><br>FastQC v0.11.9<br>BWA-MEM v2.2.1<br>Picard v3.1.1<br>Strelka v2.0.15<br>ASCAT-NGS v2.1<br>GATK 3.2-2 HaplotypeCaller<br>QDNAseq v1.4.0.0<br>ANNOVAR (April 2018)<br>dbNSFP v3. 0<br>logistf v1.25.0<br>survminer v.0.4.9<br>STAR-2.7.9a<br>GenomicRanges v1.56.1<br>SMIXnorm v0.0.0.9<br>DESeq2 v1.38.3<br>Minfi v1.50.0<br>ChAMP v2.34.0

fgsea v1.24.0
ComBat-seq
ConsensusTME v0.0.1
immunedeconv v2.1.0
SIMPLI
CellProfiler v4.2.5
Seurat v2.4
cluster v2.1.6
cMonkey2
ARACNE-AP
Network Edge Orienting (NEO)
ggpubr v0.6.0
ggplot2 v3.4.4
MSigDB v7.5.1
Reactome v.72
STRING v11.5
R v4.3.1

For manuscripts utilizing custom algorithms or software that are central to the research but not yet described in published literature, software must be made available to editors and reviewers. We strongly encourage code deposition in a community repository (e.g. GitHub). See the Nature Portfolio guidelines for submitting code & software for further information.

Data

Policy information about availability of data

All manuscripts must include a data availability statement. This statement should provide the following information, where applicable:

- Accession codes, unique identifiers, or web links for publicly available datasets
- A description of any restrictions on data availability
- For clinical datasets or third party data, please ensure that the statement adheres to our policy

DNA and RNA sequence data for the University of Cambridge (UoC) cohort were deposited at the European Genome-phenome Archive with the following accession IDs: WGS (EGAD00001011191, EGAD00001006083), shallow WGS (EGAD00001011189), bulk RNA-sequencing (EGAD00001011190). WES for 73 TCGA OACs were downloaded from the Genomic Data Commons portal (https://portal.gdc.cancer.gov/). Mutated genes for 253 Memorial Sloan Kettering Cancer Center (MSKCC) OACs that underwent targeted re-sequencing were downloaded from the cBioPortal (https://www.cbioportal.org/). Methylation data for OACs were derived from UoC (EGAD00010001822) and TCGA (https://portal.gdc.cancer.gov/). Methylation data for BOs were derived from UoC (EGAD00010001838 and EGAD00010001972). BAM files of wild type and TP53 edited CP-A cells were deposited at Zenodo (doi: 10.5281/zenodo.12918301). UoC WGS, sWGS. RNAseq and methylation data of the human patients are under controlled access by ICGC (International Cancer Consortium) due to privacy and security protection of personal data. The reasons and conditions for controlled access are described here (https://www.icgc-argo.org/page/132/data-access-and-data-use-policies-and-guidelines). The data can be accessed via the ICGC portal upon request to the ICGC Data Access Compliance Office here: https://docs.icgc-argo.org/docs/data-access/daco/applying. Source data for Figures 1-6 and Extended Data Figures 1-3 have been provided as Source Data files. All other data supporting the findings of this study are available from the corresponding author on reasonable request.

Research involving human participants, their data, or biological material

Policy information about studies with human participants or human data. See also policy information about sex, gender (identity/presentation), and sexual orientation and race, ethnicity and racism.

Reporting on sex and gender	The total cohort consisted of 1032 patients with Oesophageal Adenocarcinoma (147 Female and 885 Male) and 356 with Barrett's Oesophagus (BO) of which 257 progressed (38 Female and 219 Male) and 99 did not progress (19 Female and 80 Male) to cancer. No filtering of the human data was done based on sex or gender.
Reporting on race, ethnicity, or other socially relevant groupings	Race, ethnicity or other socially relevant groupings are not annotated and have not been considered in this study
Population characteristics	The total cohort consisted of 1032 patients with Oesophageal Adenocarcinoma (OAC) and 356 with Barrett's Oesophagus (BO) of which 257 progressed and 99 did not progress to cancer.
Recruitment	No patient was recruited specifically for this study
Ethics oversight	Written consent was obtained from all BO and OAC patients whose samples from UoC sequenced for this study (REC: 10/H0305/1 & IRAS:15757).

Note that full information on the approval of the study protocol must also be provided in the manuscript.

Field-specific reporting

Please select the one below that is the best fit for your research. If you are not sure, read the appropriate sections before making your selection.

☒ Life sciences ☐ Behavioural & social sciences ☐ Ecological, evolutionary & environmental sciences

For a reference copy of the document with all sections, see nature.com/documents/nr-reporting-summary-flat.pdf

Life sciences study design

All studies must disclose on these points even when the disclosure is negative.

Sample size	All samples with available DNA and RNA sequencing and Methylation data have been collected and used in this study with no pre-calculation of sample size. The sample size for the RNAScope-Imaging mass cytometry was chosen as the minimum number required for each of the 9p21 loss groups and 9p21 wild-type
Data exclusions	No data was excluded from the analysis
Replication	The analytical procedures implemented here are highly reproducible, consisting of fixed steps and parameters that have been described in the Methods. Each analysis was run independently by the first author and at least one of the other authors and if results did not match, it was replicated a third time. Moreover, to address one of the comments of the reviewers, we added methylation data, which required re-running the whole study. All results were replicated.<br>Growth curves of CP-A_TP53wt, CP-A_2c8, CP-A_3d2, and CP-A_5f4 cells were performed three independent times with 2-4 technical replicates each time. The quantification of CDKN2A and TP53 gene expression in these cells lines was performed once using 3 technical replicates. RNAScope and Imaging mass cytometry experiments were performed once per patient.
Randomization	The samples were split into groups according to the genomic alteration of the 9p21 locus. The study did not involve random allocation of samples.
Blinding	Blinding was not applied to this study because knowing the disease type (OAC, BO-progressor and BO-non-progressors) was integral to the analysis.

Reporting for specific materials, systems and methods

We require information from authors about some types of materials, experimental systems and methods used in many studies. Here, indicate whether each material, system or method listed is relevant to your study. If you are not sure if a list item applies to your research, read the appropriate section before selecting a response.

Materials & experimental systems

n/a	Involved in the study	
☐	☒	Antibodies
☐	☒	Eukaryotic cell lines
☒	☐	Palaeontology and archaeology
☒	☐	Animals and other organisms
☒	☐	Clinical data
☒	☐	Dual use research of concern
☒	☐	Plants

Methods

n/a	Involved in the study	
☒	☐	ChIP-seq
☒	☐	Flow cytometry
☒	☐	MRI-based neuroimaging

Antibodies

Antibodies used

Antibody (clone) Dilution Vendor Catalogue#
p16 (F-12) 1:150 Santa Cruz SC-1661
MTAP (2G4) 1:200 Novus Biologicals H00004507-M01
Digoxinenin (IFNE) -611621 1:500 R&D Systems MAB7520
IFNA2 (Polyclonal) 1:500 Abcam Ab198914
IFNA4 (Polyclonal) 1:100 Abcam Ab230843
IFNW1 (Polyclonal) 1:800 Invitrogen PA5-96942
Biotin (IFNB1) (1D4-C5) 1:500 Biolegend 409002
Vimentin (RV202) 1:300 Standard Biotools 3143029D
Cadherin-1 (24E10) 1:3000 Standard Biotools 3158029D
Pan-keratin (C11) 1:2000 Standard Biotools 3148020D
GzmB (EPR20129-217) 1:300 Standard Biotools 3167021D
Ki67 (B56) 1:400 Standard Biotools 3168022D
FOXP3 (236A/E7) 1:200 Standard Biotools 3155016D
CD4 (EPR6855) 1:200 Standard Biotools 3156033D
CD8 (D8A8Y) 1:800 Standard Biotools 3162035D
CD3 (Polyclonal) 1:800 Standard Biotools 3170019D
CD27 (EPR8569) 1:300 Standard Biotools 3171024D
HLA-DR/DP/DQ (CR3/43) 1:500 Abcam ab7856
CD11b (D6X1N) 1:1000 Cell Signalling 49420
CD163 (EDHu-1) 1:300 Standard Biotools 3147021D
CD68 (KP1) 1:400 Standard Biotools 3159035D
CD14 (EPR3653) 1:200 Standard Biotools 91H003153
CD15 (W6D3) 1:250 Standard Biotools 3149026D

CD16 (EPR16784) 1:200 Standard Biotools 91H004148
NCAM1 (EP2567Y) 1:250 Abcam Ab214435
CD11c (EP1347Y) 1:400 Abcam Ab216655
FITC (PPIB) (FIT-22) 1:100 Standard Biotools 3144006B

Validation

All antibodies are commercially available and validated:

All Standard Biotools antibodies used in this study are Maxpar® antibodies. Antibodies for vimentin, cadherin-1, pan-keratin, granzyme B (GzmB), Ki67, FOXP3, CD4, CD8, CD3, CD27, CD163, CD68, CD14, CD15, CD16 were developed and optimized for use with the Hyperion™ Imaging System with formalin-fixed paraffin-embedded (FFPE) human tissue sections. The antibody for fluorescein (FITC) (3144006B) was validated in-house for use for Hyperion Imaging System (Montorsi et al., 2023). Each lot of metal-conjugated antibody was quality-control-tested on human oesophageal cancer FFPE tissue sections using the Hyperion Imaging System. The staining patterns of all markers were verified by an independent pathologist.
Antibodies for HLA-DR/DP/DQ, CD11b and CD11c had already been conjugated to their metal isotopes in-house for another study (Bortolomeazzi et al., 2021). They were tested on human oesophageal adenocarcinoma FFPE tissue sections using Hyperion Imaging System with the dilutions reported by (Bortolomeazzi et al., 2021). The dilutions for HLA-DR/DP/DQ, CD11b and CD11c were 1:500, 1:1000, and 1:400, respectively.

Antibodies for p16, MTAP, IFNA2, IFNW1 and NCAM1 were validated by immunohistochemistry on human oesophageal adenocarcinoma and human colorectal cancer FFPE tissue sections. The antibody for IFNA4 was validated by immunohistochemistry in human oesophageal adenocarcinoma and human tonsil FFPE tissue sections. Staining was done using the Mouse and Rabbit Specific HRP/DAB (ABC) Detection IHC kit (ab64264) following the instructions of the manufacturer. Heat-mediated antigen retrieval was performed using Tris/EDTA buffer (pH 9.0). Antibodies were diluted in SuperBlock™ Blocking Buffer from ThermoFisher Scientific™ with the following dilutions: 1:600, 1:400, 1:2000, 1:5000, 1:5000 and 1:400, respectively. Tissues were counterstained with Haematoxylin. These antibodies were then conjugated to their metal isotopes in-house using the Maxpar® X8 metal conjugation kit (Standard Biotools). After conjugation, they were tested by RNAScope coupled to Hyperion Imaging System at three different dilutions. For each antibody, the dilution with the highest signal-to-noise ratio was chosen for the final experiment (Table S9).

RNAScope Fluorescent Multiplex Assay for UBC, PPIB and POLR2 (positive control probes) was performed on human oesophageal adenocarcinoma FFPE tissue sections to check for their expected expression and staining pattern. Antibodies targeting biotin, digoxigenin and FITC were then validated by RNAScope™ coupled to Hyperion Imaging System in human oesophageal adenocarcinoma FFPE tissue sections using the RNAScope probes for UBC, PPIB and POLR2. The dilutions for these antibodies had already been determined by (Montorsi et al., 2023).

References
Bortolomeazzi, M. et al. Immunogenomics of Colorectal Cancer Response to Checkpoint Blockade: Analysis of the KEYNOTE 177 Trial and Validation Cohorts. Gastroenterol 161, 4, doi: 10.1053/j.gastro.2021.06.064 (2021).

Montorsi, L. et al. Unanticipated interacting features of human gut-associated lymphoid tissues link microbiota, intestinal immunity and autoimmunity. bioRxiv doi: https://doi.org/10.1101/2023.08.29.555265 (2023)

Eukaryotic cell lines

Policy information about cell lines and Sex and Gender in Research

Cell line source(s)

The human Barrett's oesophagus cell line called CP-A (derived from an adult male with metaplastic BO)(Palanca-Wessels, 2003) used in this study was obtained from the Francis Crick Institute Cell Services facility (ATCC catalogue number CRL-4027).

Authentication

Method: For Cell Authentication, the Cell Services team at the Francis Crick Institute used STR (Short Tandem Repeat) Profiling for the CP-A cell line using the Promega PowerPlex16HS system. This profile was compared back to the profile available in ATCC. The species was then confirmed using a primer system based on the Cytochrome C Oxidase Subunit 1 gene from mitochondria.
Result: The STR profile compares with ATCC except it shows 11,14 at D13S317 and not 12,14
Date: 14/08/17

Mycoplasma contamination

Method: For Mycoplasma screening two different tests were used– Agar Culture (which involves culturing any mycoplasma that may be present in the cell culture on specialised agar) and Fluorescent staining using the Hoescht Stain.
Result: The cell-line is free of mycoplasma contamination
Date: 14/08/17

Commonly misidentified lines
(See ICLAC register)

CP-A cell line is not listed under commonly misidentified lines

Plants

Seed stocks	*Report on the source of all seed stocks or other plant material used. If applicable, state the seed stock centre and catalogue number. If plant specimens were collected from the field, describe the collection location, date and sampling procedures.*
Novel plant genotypes	*Describe the methods by which all novel plant genotypes were produced. This includes those generated by transgenic approaches, gene editing, chemical/radiation-based mutagenesis and hybridization. For transgenic lines, describe the transformation method, the number of independent lines analyzed and the generation upon which experiments were performed. For gene-edited lines, describe the editor used, the endogenous sequence targeted for editing, the targeting guide RNA sequence (if applicable) and how the editor was applied.*
Authentication	*Describe any authentication procedures for each seed stock used or novel genotype generated. Describe any experiments used to assess the effect of a mutation and, where applicable, how potential secondary effects (e.g. second site T-DNA insertions, mosiacism, off-target gene editing) were examined.*
