# Supplementary figures and images for: Context-dependent effects of CDKN2A and other 9p21 gene losses during the evolution of esophageal cancer
Source: Nat Cancer. 2025 Jan 3;6(1):158–74. doi: 10.1038/s43018-024-00876-0 (PMC11779637; doi:10.1038/s43018-024-00876-0)

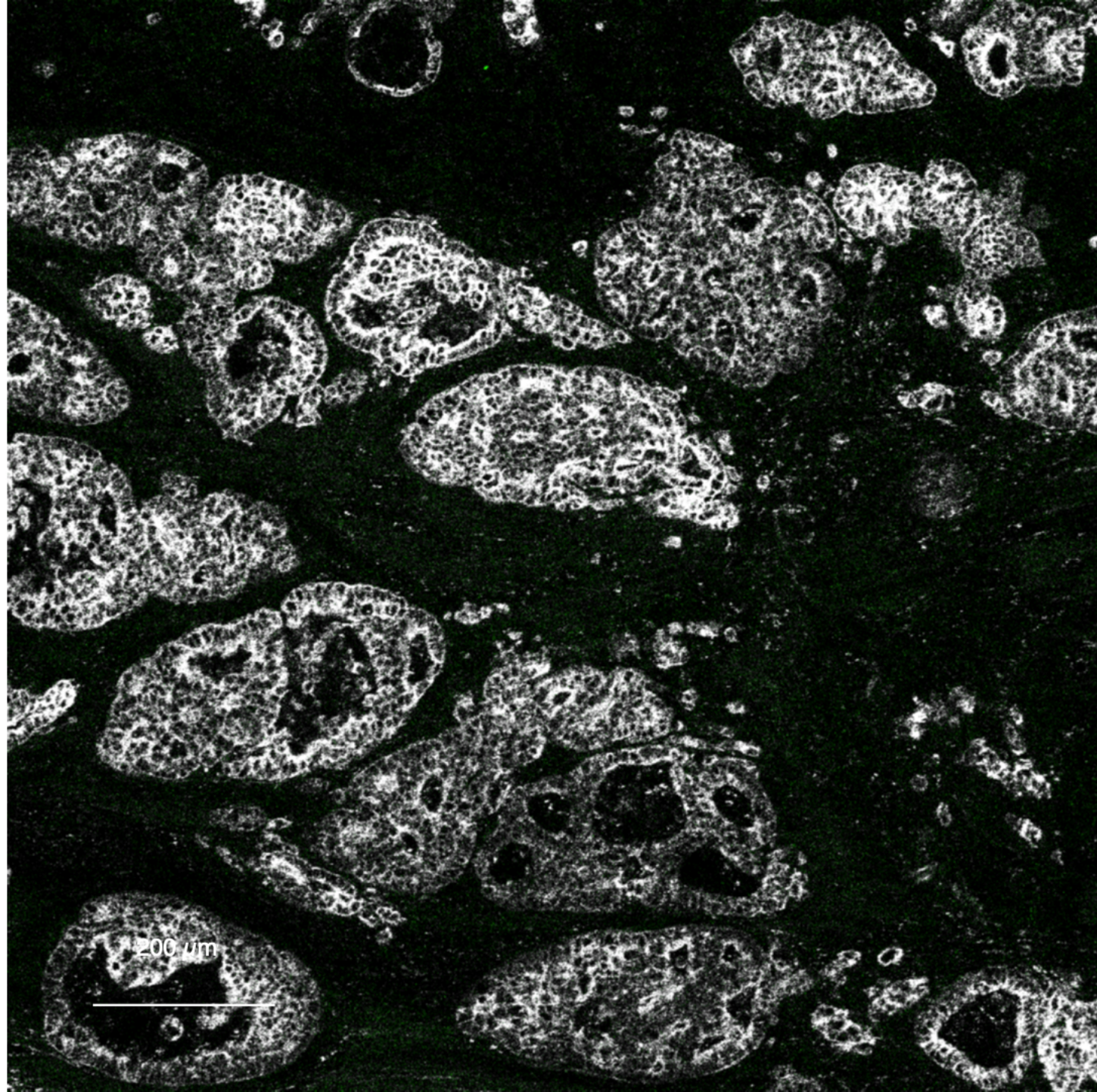

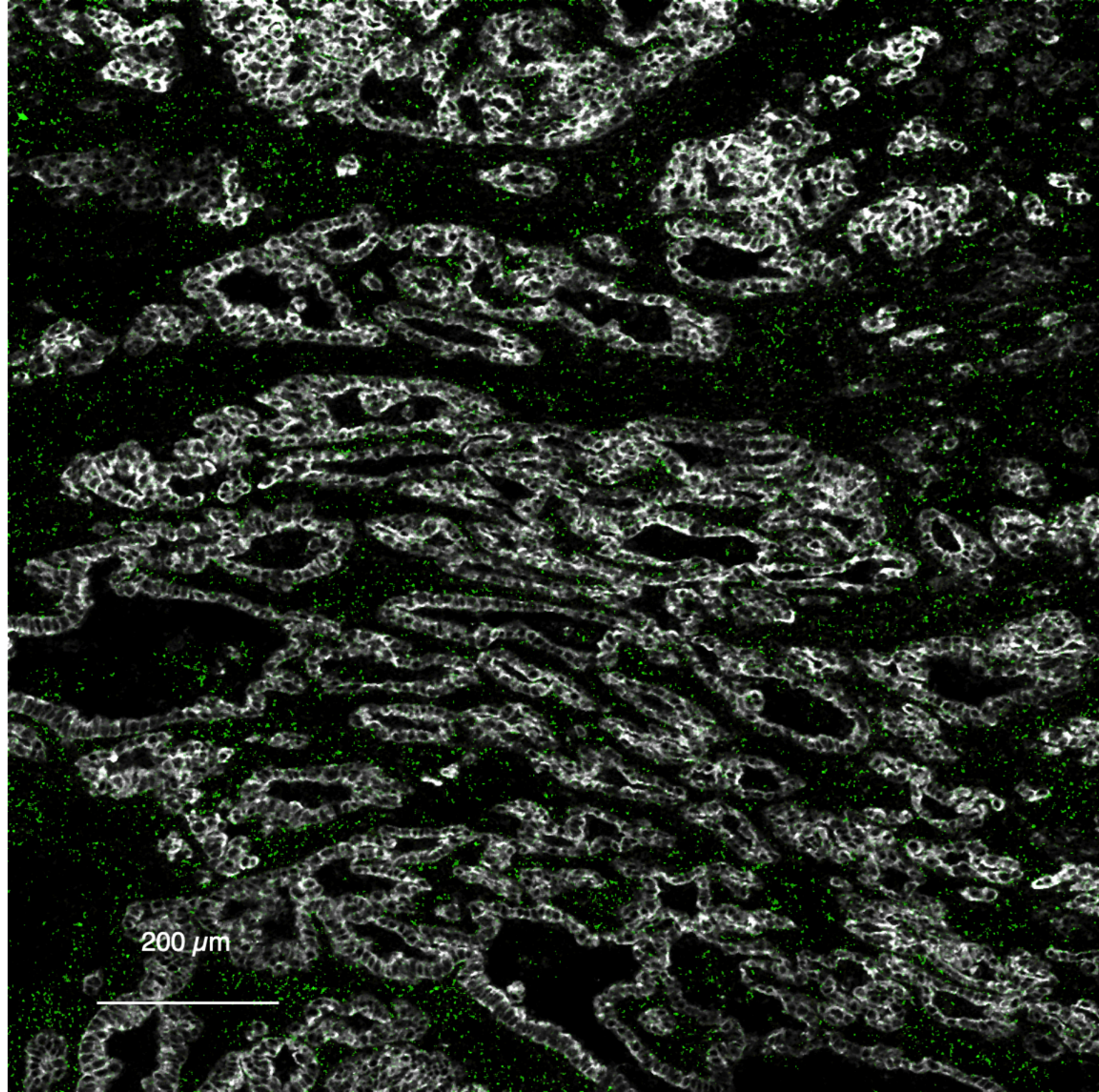

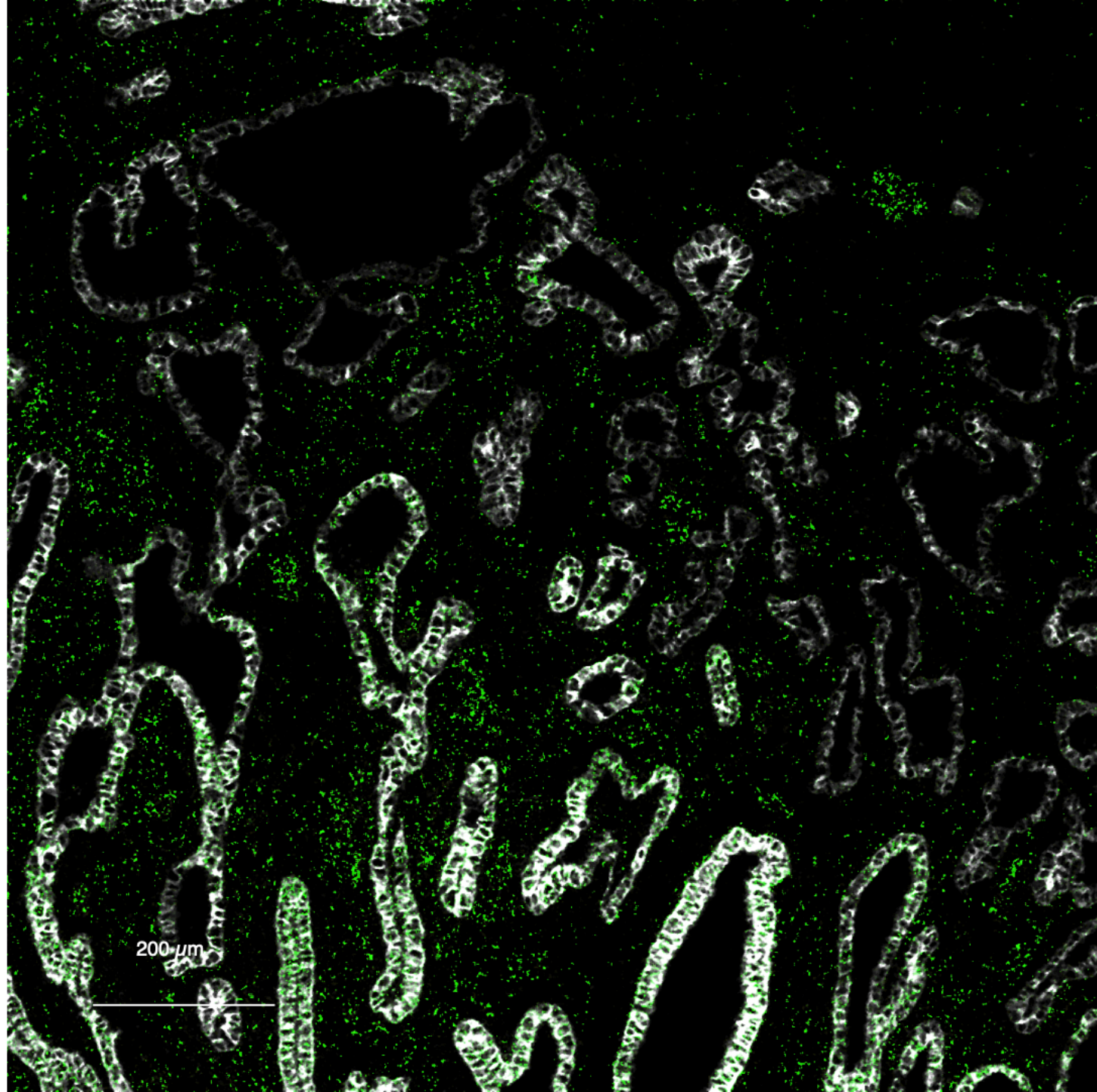

Supplement: Supplementary file 8 — IMC images. [file 43018_2024_876_MOESM8_ESM.pdf]
